# Faint Lines in the Arc Spectrum of Iron (Fe I)

**DOI:** 10.6028/jres.065A.001

**Published:** 1961-02-01

**Authors:** Carl C. Kiess, Vera C. Rubin, Charlotte E. Moore

## Abstract

A search for new faint iron lines has been made on spectrograms taken with an arc-in-air as source. The range of observations is from 2102 to 8679 A. The reciprocal dispersion of the spectrographs used for the various spectral regions varies from 1 A/mm to 3 A/mm.

Twelve new energy levels have been found, resulting in a total of 121 classified lines. A table containing 698 classified lines includes many lines whose wavelengths had been predicted as combinations among the known energy levels, and found in the solar spectrum in earlier work. Their reality has been confirmed in the present work.

A list containing 1,102 newly measured unclassified lines is included. Many of the lines listed in the tables have been reported by other observers with varying degrees of accuracy. All such reference sources are indicated in the tables. As a result of the new measurements, these lines may safely be attributed to Fe I.

A comparison of the new lines with the solar spectrum has resulted in the identification of 306 solar lines of Fe I unblended, and of 85 as blends to which Fe I is a contributor.

When the analysis of Fe I was carried out in 1944,[1],^1^ it was recognized that the laboratory observations were far from complete. From the known atomic energy levels, wavelengths were calculated for missing members of multiplets. The presence in the solar spectrum of almost all known lines of neutral iron indicated that a search for these “predicted” lines might be rewarding. The predicted lines were graded into three classes, good, fair, and poor, the grades being assigned roughly on the likelihood of the transition according to the rules of the quantum theory, the agreement between calculated and solar wavelengths, and the solar intensity with respect to known lines in the respective multiplets. The lines in the categories “good” and “fair” were published in Table C of the 1944 Monograph [1].

A search for faint iron lines has been made on spectrograms taken with an arc-in-air as source, and with exposures long enough to reveal fainter lines than have been recorded previously. The range of observations is from 2102 to 8679 A. The electrodes used in the arcs were prepared from the purest iron obtainable at the time. The observations were made by the senior author (CCK) with the spectrographs at NBS. The spectrograms that have been measured are listed in table 1. The letters indicate the type of instrument used to cover the various spectral regions, namely:
HPrism: reciprocal dispersion 1 A/mm to 1/3 A/mm.XGrating: Wood, 21 ft. concave grating 30,000 lines per inch, reciprocal dispersion 1 A/mm in the second order.RGrating: Rowland, 21 ft. concave grating 20,000 lines per inch, reciprocal dispersion 3 A/mm.

In order to obtain at least two exposures over the entire range it has been necessary to fill in gaps by using Fe comparison spectra from miscellaneous spectrograms in the collection at the Georgetown College Observatory. These were taken with the Rowland grating described above. A serious attempt has been made to eliminate all possible impurities from the spectra.

The present work has been carried out to confirm the predicted lines attributed to Fe I in the solar spectrum, and to extend the identifications of Fe I in the sun. It was hoped, also, that the analysis could be extended by additional observations. A search has been made among the new unclassified lines for levels combining with the known low terms of Fe I. Twelve new levels have been found, of which three are subject to some question. These levels are listed in table 2 with their respective *J*-values entered in column 2. They account for a total of 121 classified lines. Observations with a more suitable source will be required to extend this study further. One serious disadvantage encountered here is the masking of faint lines by wings of stronger ones. An electrodeless lamp doubtless would excite many new faint lines, and thus make it possible to find additional terms and assign their configurations.

The classified lines are listed in table 3. The wave lengths in column 1 are the mean values from the present work. They include early unpublished measurements by Burns and Kiess, as well as a number of more recent ones by various workers. In preparing the final lists no line has been included as real unless it has been measured on at least two exposures. Many have more than two measurements. The classifications are new except for those lines in Table C of the Monograph that have been confirmed in the present work, and those lines having notes (a) and (b).

Some wavelengths are in italics. These were included in the original Princeton line list as unclassified and have been used for combinations with the new levels in Table 2. The best available reference source has been adopted for these lines, as was done in the Monograph. They are not new lines, but are newly classified.

The total number of lines in table 3 is 698. The lines are mostly faint. Estimated intensities on an arbitrary scale are given in column 2. Diffuse lines are indicated by *“n*” and *“N”.* The next two columns contain, respectively, the observed wave numbers and those calculated from the term combinations. The designations in column 5 are those used for Fe I in the 1944 Monograph [1] and in “Atomic Energy Levels” [2]. The multiplet numbers in column 6 are from the 1945 Princeton Multiplet Table [3]. New lines belonging to known multiplets have been assigned the appropriate multiplet number. The notes in column 7 explain the different categories of lines. Most of the classifications are either “New” or “Pred”. The latter include lines from Table C of the Monograph, i.e., those predicted lines classed as “good” or “fair” for which a solar wavelength was used. Additional predicted lines are also included, as indicated by the note (b) in this column. Although the predicted Fe I lines were classified earlier, they are included because for the first time they have been confirmed from laboratory observations. A few lines have the note “SS”. The solar wavelengths were previously used for these lines as preferable to various laboratory values.

The letters in the last column are the same ones used for reference sources in the Monograph line list. They refer to lines that are not new but are newly classified, to lines measured by earlier observers that needed additional confirmation, or to unpublished measurements from the Massachusetts Institute of Technology, kindly furnished by G. R. Harrison in 1942. References Z, BK, and ZZ have been added.

In 1934 a list of faint lines of Fe I was compiled by Burns and Kiess. It contains early unpublished measurements made by Burns at Bonn and possibly at the Allegheny Observatory, and, also, measurements by Kiess of “H” plates described above. Lines in tables 3 and 4 that are present in this early list are so indicated by “BK” in the last column.

Table 4 contains the newly measured unclassified lines of Fe I, 1102 in all. The four columns contain respectively, the wavelengths, estimated intensities, wave numbers, and a column headed “Notes and References”. As before, diffuse lines are marked *“n”* and “*N*” in the intensity column. In the last column the letters denote the same reference sources as were used in the 1944 paper. The symbols and references are described at the end of table 3.

Table 5 contains the solar lines that have been newly identified as Fe I, from the present work. The total number is 391, of which 306 are unblended, and 85 blended in the solar spectrum. The left-hand part of the table contains the laboratory data, i.e., wavelength and intensity from tables 3 and 4. The solar data are entered in the right-hand part of the table: wavelength, disk intensity, the difference between the solar and laboratory wavelength, (⊙—lab.), and the solar identification. These data are from the current revision of the solar spectrum now in progress [5]. From X 2945 to λ 3164 the disk intensities are eye estimates: those in the range λ 2945 to the λ 3062 are from the 1948 paper [6], from λ 3062 to λ 3164 they are from Rowland’s Preliminary Table of Solar Spectrum Wavelengths, as quoted in the 1928 edition [4]. The estimated intensities are entered in brackets. From λ 3164 to longer waves Rowland’s estimated intensities are replaced by equivalent widths measured by Minnaert and Houtgast at Utrecht [5]. Italics denote that the reduced equivalent width is the weighted mean of the Utrecht measurements and of those by other observers.

In the last column of table 5 a predominant contributor to a solar line that is a blend, is indicated by the symbol “║”. A leading contributor has the symbol “|”. A dash is used in this column to show whether the contributors to a blend are on the short - or long-wave side of the solar line. For example, the solar line at 4424.072 A is identified as a blend of Fe I on the short-wave side and Cr 1 on the longwave side. Approximately 50 more lines in the present lists, whose identification in the solar spectrum is more dubious, have been omitted from table 5.

In conclusion, it is a pleasure to express our gratitude to those who have assisted with this laborious program. Special thanks are due Misses Eva Novotny and Janet Rountree for their work in measuring the plates. Mrs. Isabel Murray has prepared the tabular data with meticulous care. The work could not have been brought to its present stage of completion without special financial aid. The project has been carried in part by grants NR 046–136 from the Office of Naval Research and G 8193 from the National Science Foundation to the Georgetown College Observatory. Both of these are gratefully acknowledged.

## Figures and Tables

**Table 1:** List of spectrograms

Plate	Region A	Plate	Region A
			
H 53	2102 to 2178	X 242	3007 to 3773
H 55	2103 to 2178	X 441	3090 to 3820
H 41	2136 to 2224	X 546	3348 to 3631
H 42	2138 to 2231	X 430	3358 to 4482
H 27	2184 to 2301	X 436	3581 to 3820
H 26	2184 to 2303	X 244	3614 to 4294
H 13	2260 to 2368	X 132	4250 to 4635
H 1	2327 to 2474	X 275	4280 to 4425
H 65	2404 to 2582	X 303	4400 to 6625
H 76	2488 to 2714	X 630	4632 to 5216
H 86	2611 to 2851	X 453	4752 to 6421
H 96	2767 to 3083	X 304	5145 to 6253
		X 452	6885 to 8643
		R 640	6608 to 7945
		R 638	7832 to 8679

**Table 2:** New odd levels of *Fe I*

Level	*J*	Level	*J*
			
*49457.36*°?	4	*53881.91*°	4
*53357.53*°	3	*54289.09*°	3
*53610.44*°	4	*54357.40*°	3
*53733.51*°	3	*57565.35*°?	3
*53749.39*°	2	*60563.61*°	3
*53784.74*°	3	*62081.27*°?	2

**Table 3:** Classified faint lines of *Fe I*

Wavelength A	Intensity	Wave number (cm^−1^)		Designation	Multiplet Number	Notes	Reference
		Observed	Calc.				
							
*10400.94*	1	9611.88	2.08	$e{\;}^{5}D_{4}-54289_{3}^{0}$		New	D
8656.702	1	11548.57	8.63	*x* ${\;}^{5}D_{0}^{{}^{\circ}}$—*e* ^3^D_1_	1269	Pred	U
8616.275	2*n*	11602.76	2.77	*x* ${\;}^{5}D_{4}^{{}^{\circ}}$—*e* ^7^G_5_	1266	Pred	
8571.827	1*n*	11662.92	2.91	*x* ${\;}^{5}D_{1}^{{}^{\circ}}$—*e* ^5^P_2_	1272	Pred	
8562.138	1*n*	11676.12	6.14	*z* ${\;}^{3}G_{3}^{{}^{\circ}}$—*e* ^5^F_3_	1153	Pred	U
8538.016	1	11709.11	9.11	*x* ${\;}^{5}D_{4}^{{}^{\circ}}$*—e* ^7^G_4_	1266	Pred	
8481.992	1	11786.44	6.50	$e{\;}^{3}F_{2}-x{\;}^{3}D_{1}^{{}^{\circ}}$	999	Pred	
8446.394	3	11836.12	6.09	*x* ${\;}^{5}D_{2}^{{}^{\circ}}$*—e* ^5^P_2_	1272	Pred^b^	BK
8434.504	1*n*	11852.80	2.79	*x* ${\;}^{5}D_{1}^{{}^{\circ}}$*—g* ^5^D_0_	1270	Pred	U
8358.512	1	11960.56	0.54	$b{\;}^{3}G_{4}-z{\;}^{3}G_{3}^{{}^{\circ}}$	401	Pred	U
8300.006	1	12044.87	4.87	$X_{3}-v{\;}^{3}P_{2}^{{}^{\circ}}$	1331	Pred	
8269.663	1	12089.07	9.08	$d{\;}^{3}F_{4}-v{\;}^{3}D_{3}^{{}^{\circ}}$	1218	Pred	
8231.749	2	12144.75	4.75	*x* ${\;}^{5}D_{4}^{{}^{\circ}}$—*g* ^5^D_3_	1270	New	
8204.959	1	12184.40	4.44	$a{\;}^{5}F_{3}-z{\;}^{7}D_{2}^{{}^{\circ}}$	12	Pred	U
8196.492	1	12196.99	6.95	$d{\;}^{3}F_{4}-u{\;}^{3}F_{3}^{{}^{\circ}}$	1217	Pred	
8126.520	1	12302.01	2.08	$d{\;}^{3}F_{2}-v{\;}^{3}D_{2}^{{}^{\circ}}?$	1218	New	
8112.178	2	12323.76	3.77	$a{\;}^{3}G_{5}-y{\;}^{5}F_{4}^{{}^{\circ}}$	265	Pred	U
8108.344	1	12329.58	9.61	$a{\;}^{3}G_{4}-y{\;}^{5}F_{3}^{{}^{\circ}}$	265	Pred	U
8090.341	1	12357.02	7.06	$d{\;}^{3}F_{2}-v{\;}^{3}D_{1}^{{}^{\circ}}?$	1218	New	
8027.667	2	12453.03	3.04	$a{\;}^{3}D_{3}-y{\;}^{3}D_{2}^{{}^{\circ}}$	623	Pred	
8002.586	1	12492.52	2.58	$d{\;}^{3}F_{2}-w{\;}^{3}F_{2}^{{}^{\circ}}$	1217	Pred	
7941.810	1	12588.12	8.08	$a{\;}^{1}G_{4}-y{\;}^{3}F_{3}^{{}^{\circ}}$	508	Pred	
7924.184	1	12616.12	6.20	*y* ${\;}^{3}D_{2}^{{}^{\circ}}$*—e* ^3^D_3_	1250	Pred	U
7810.836	0*n*	12799.20	9.25	*x* ${\;}^{5}F_{4}^{{}^{\circ}}$—*g* ^5^F_4_	1303	Pred	
7808.004	2*n*	12803.85	3.91	*x* ${\;}^{5}F_{5}^{{}^{\circ}}$—*g* ^5^F_5_	1303	Pred	BK, O, Z
7745.496	0	12907.18	7.20	*x* ${\;}^{5}F_{2}^{{}^{\circ}}$—*f* ^5^P_1_	1305	Pred	
7719.064	1	12951.37	1.41	*x* ${\;}^{5}F_{4}^{{}^{\circ}}$—*h* ^5^D_3_	1304	Pred	
7650.948	1	13066.68	6.68	$a{\;}^{3}G_{5}-z{\;}^{5}G_{5}^{{}^{\circ}}$	266	New	
7647.850	0	13071.97	2.00	*z* ${\;}^{5}G_{2}^{{}^{\circ}}$*—e* ^3^F_2_	1137	Pred	U
7617.984	0	13123.22	3.25	$c{\;}^{3}F_{2}-u{\;}^{5}D_{2}^{{}^{\circ}}$	1001	Pred	
7617.242	0	13124.50	4.59	*x* ${\;}^{5}F_{3}^{{}^{\circ}}$—*h* ^5^D_2_	1304	Pred	
7606.460	0	13143.10	3.12	*x* ${\;}^{5}F_{2}^{{}^{\circ}}$—*f* ^5^G_3_	1306	New	
7588.287	1	13174.58	4.55	*x* ${\;}^{5}F_{4}^{{}^{\circ}}$—*f* ^5^G_4_	1306	Pred	U
7547.902	0	13245.07	5.10	*x* ${\;}^{5}F_{1}^{{}^{\circ}}$—*f* ^5^G_2_	1306	Pred	U
7540.415	0	13258.22	8.18	$a{\;}^{3}G_{4}-z{\;}^{5}G_{4}^{{}^{\circ}}$	266	Pred	U
7537.531	0	13263.29	3.46	$c{\;}^{3}F_{4}-w{\;}^{5}P_{3}^{{}^{\circ}}$	1000	Pred^b^	
7476.378	1	13371.78	1.75	*y* ${\;}^{3}D_{2}^{{}^{\circ}}$—*g* ^5^D_2_	1251	SS	O, U
7382.670	1	13541.50	1.58	$a{\;}^{3}G_{5}-z{\;}^{5}G_{4}^{{}^{\circ}}$	266	Pred	U
7359.950	1	13583.31	3.31	*x* ${\;}^{5}F_{5}^{{}^{\circ}}$—*e* ^3^H_6_	1310	Pred	
7344.171	1	13612.49	2.48	$a{\;}^{3}G_{4}-z{\;}^{5}G_{3}^{{}^{\circ}}$	266	Pred	
7330.148	1	13638.53	8.52	*y* ${\;}^{5}P_{1}^{{}^{\circ}}$—*f* ^7^D_1_	1187	Pred	U
7317.402	1	13662.29	2.28	*x* ${\;}^{5}D_{1}^{{}^{\circ}}$—*f* ^3^D_2_	1278	Pred	
7316.752	1	13663.50	3.47	*a* ^3^G_5_—*z* ${\;}^{3}G_{5}^{{}^{\circ}}$	267	Pred	U
7315.595	1	13665.66	5.68	*z* ${\;}^{3}P_{0}^{{}^{\circ}}$—*e* ^5^F_1_	1105	New	
7300.532	1*n*	13693.86	3.76	*c* ^3^F_3_—*z* ${\;}^{3}H_{4}^{{}^{\circ}}$	1003	Pred	U
7256.180	1*n*	13777.56	7.67	*x* ${\;}^{5}D_{3}^{{}^{\circ}}$—*f* ^3^D_3_	1278	SS	U
7213.900	0	13858.31	8.43	*z* ${\;}^{3}P_{1}^{{}^{\circ}}$*—e* ^5^F_1_	1105	Pred	
7197.182	1	13890.50	0.51	*y* ${\;}^{5}P_{2}^{{}^{\circ}}$—*f* ^7^D_1_	1187	New	
7190.143	1	13904.10	4.14	*c* ^3^P_0_— *y* ${\;}^{3}D_{1}^{{}^{\circ}}$	463	Pred	
7127.576	1*n*	14026.15	6.16	*x* ${\;}^{5}D_{2}^{{}^{\circ}}$*—g* ^5^F_2_	1273	Pred	U
7119.987	1	14041.10	1.06	*y* ${\;}^{5}P_{3}^{{}^{\circ}}$—*f* ^7^D_4_	1187	Pred	
7118.106	1*n*	14044.81	4.79	*x* ${\;}^{5}D_{1}^{{}^{\circ}}$*—f* ^3^D_1_	1278	Pred	
7114.527	1*n*	14051.87	1.82	*a* ^3^G_5_—*z* ${\;}^{3}G_{4}^{{}^{\circ}}$	267	Pred	
7093.042	1*n*	14094.44	4.32	*y* ${\;}^{5}P_{3}^{{}^{\circ}}$*—e* ^7^P_2_	1189	Pred	O, U
7092.866	1*n*	14094.79	4.85	*y* ${\;}^{5}P_{3}^{{}^{\circ}}$*—f* ^7^D_3_	1187	New	U
7072.832	1	14134.71	4.75	*c* ^3^F_4_—*z* ${\;}^{3}H_{5}^{{}^{\circ}}$	1003	Pred	
7069.571	0	14141.23	1.30	*b* ^3^F_4_—*z* ${\;}^{5}G_{5}^{{}^{\circ}}$	205	SS	U
6936.501	1	14412.52	2.57	*y* ${\;}^{5}P_{2}^{{}^{\circ}}$*—e* ^7^S_3_	1196	Pred	U
6847.605	1	14599.62	9.65	*y* ${\;}^{5}F_{3}^{{}^{\circ}}$*—e* ^3^F_2_	1078	SS	U
6785.754	1	14732.69	2.69	*d* ^3^F_3_—*y* ${\;}^{1}D_{2}^{{}^{\circ}}$	1226	Pred	
6745.969	1	14819.58	9.61	*c* ^3^F_4_—*w* ${\;}^{5}G_{3}^{{}^{\circ}}$	1005	Pred	
6704.513	1	14911.21	1.30	*y* ${\;}^{5}D_{1}^{{}^{\circ}}$*—e* ^3^F_2_	1052	SS	U
6565.700	1*n*	15226.46	6.39	X_3_—*s* ${\;}^{3}G_{3}^{{}^{\circ}}$?		New	
6249.667	2*n*	15996 43	6.49	*z* ${\;}^{5}F_{4}^{{}^{\circ}}$—*e* ^7^D_4_	685	Pred	
*6028.344*	1	16583.71	3.68	*c* ^3^F_4_— $49457_{4}^{{}^{\circ}}$	685	New	U
*5905.030*	1	16930.02	0.07	*e* ^7^D_2_— $60564_{3}^{{}^{\circ}}$		New	U
5899.094	2	16947.06	7.22	*a* ^1^D_2—_*x* ${\;}^{3}D_{1}^{{}^{\circ}}$	738	New	
5738.248	3	17422.09	2.17	*y* ${\;}^{5}F_{4}^{{}^{\circ}}$—*f* ^5^F_4_	1084	Pred	
5473.171	2	18265.87	5.85	*y* ${\;}^{5}D_{2}^{{}^{\circ}}$*—e* ^5^P_2_	1064	Pred	BK
5470.105	3	18276.11	6.06	*z* ${\;}^{5}G_{2}^{{}^{\circ}}$*—h* ^5^D_1_	1144	(a)	W
5438.024	3	18383.92	3.89	*d* ^3^F_4_—*v* ${\;}^{3}H_{5}^{{}^{\circ}}$	1237	Pred	
5407.401	3	18488.03	8.17	*a* ^1^D_2_—*x* ${\;}^{3}F_{3}^{{}^{\circ}}$?		New	
5406.790	3	18490.12	0.20	*z* ${\;}^{5}G_{4}^{{}^{\circ}}$—*f* ^3^D_3_	1148	Pred	
5401.260	3	18509.05	9.04	*z* ${\;}^{5}G_{6}^{{}^{\circ}}$*—e* ^5^H_6_	1146	Pred	
5308.678	1	18831.84	1.75	*y* ${\;}^{5}F_{3}^{{}^{\circ}}$—*f* ${\;}^{5}P_{3}^{{}^{\circ}}$	1091	Pred	
5305.397	0	18843.49	3.44	*b* ^3^D_1_—*v* ${\;}^{5}P_{2}^{{}^{\circ}}$	877	Pred^b^	
5300.403	1	18861.24	1.22	*d* ^3^F_4_—*s* ${\;}^{3}G_{5}^{{}^{\circ}}$	1240	Pred	
5297.142	2	18872.85	2.81	*b* ^3^G_4_—*z* ${\;}^{5}H_{5}^{{}^{\circ}}$	407	New	
5245.717	3	19057.87	7.88	*a* ^1^P_1_—*z* ${\;}^{3}S_{1}^{{}^{\circ}}$	715	Prod	
5238.246	2	19085.05	5.04	*z* ${\;}^{3}F_{2}^{{}^{\circ}}$—*e* ^5^G_3_	962	Prod	
5234.594	3	19098.36	8.54	*b* ^3^H_4_—*x* ${\;}^{5}G_{5}^{{}^{\circ}}$		New c	BK
*5221.046*	1	19147.92	7.92	*b* ^1^D_2_— $53785_{3}^{{}^{\circ}}$		New	U
5214.616	3	19171.53	1.55	*b* ^1^D_2_*—v* ${\;}^{3}P_{1}^{{}^{\circ}}$	1131	New	
5213.827	1	19174.43	4.53	*z* ${\;}^{3}F_{3}^{{}^{\circ}}$—*e* ^5^G_4_	962	Pred	
5211.216	2	19184.04	4.14	*a* ^1^P_1_*—y* ${\;}^{3}P_{2}^{{}^{\circ}}$	716	New	
5209.883	3	19188.95	8.91	*b* ^3^H_6_*—y* ${\;}^{3}G_{5}^{{}^{\circ}}$	584	Pred	
5207.960	3	19196.03	6.10	*b* ^3^D_1_*—x* ${\;}^{3}P_{1}^{{}^{\circ}}$	880	SS	W, ZZ
5205.372	1	19205.58	5.60	*b* ^3^H_4_—*x* ${\;}^{5}G_{4}^{{}^{\circ}}$		New	
5204.953	2	19207.12	7.13	*b* ^3^G_4_—*z* ${\;}^{5}H_{3}^{{}^{\circ}}$	407	Pred^b^	
5197.942	3	19233.03	3.09	*y* ${\;}^{5}F_{1}^{{}^{\circ}}$—*f* ^5^P_1_	1091	Pred	U
5174.703	2	19319.40	9.56	*c* ^3^P_0_—*w* ${\;}^{5}D_{1}^{{}^{\circ}}$?	465	New	
5157.156	3*n*	19385.13	5.25	X_2_— $60564_{3}^{{}^{\circ}}$		New	U, W
5156.599	2	19387.23	7.22	*z* ${\;}^{3}F_{3}^{{}^{\circ}}$*—e* ^7^F_4_	960	New	
5149.492	1	19413.98	3.96	*z* ${\;}^{3}F_{3}^{{}^{\circ}}$*—e* ^5^G_3_	962	New	
5146.322	3	19425.94	6.05	*z* ${\;}^{5}G_{4}^{{}^{\circ}}$—*f* ^3^F_4_	1150	Pred	BK
5143.740	2*n*	19435.69	5.75	*a* ^5^P_2_—*y* ${\;}^{3}F_{3}^{{}^{\circ}}$	65	Pred b	
5123.284	3*n*	19513.29	$\left\{ \;\begin{array}{l} 3.41\\ 3.32 \end{array}\right.$	*a* ^3^D_2_— *w* ${\;}^{5}P_{3}^{{}^{\circ}}$ *z* ${\;}^{5}G_{3}^{{}^{\circ}}$—*f* ^3^F_3_	629 1150	New Pred	} BK, U
5091.722	2	19634.25	$\left\{ \;\begin{array}{l} 4.29\\ 4.25 \end{array}\right.$	*a* ^1^D_2_—*v* ${\;}^{5}F_{2}^{{}^{\circ}}$ *a* ^1^P_1_—*u* ${\;}^{5}D_{1}^{{}^{\circ}}$	745 717	Pred b Pred	
5088.164	3	19647.98	7.99	*y* ${\;}^{5}D_{3}^{{}^{\circ}}$—*h* ^5^D_4_	1066	SS	ZZ
5084.549	1	19661.95	1.96	*b* ^1^G_4_—*v* ${\;}^{3}G_{5}^{{}^{\circ}}$	932	Pred	
5080.928	3	19675.96	5.89	*b* ^3^H_5_*—z* ${\;}^{3}I_{6}^{{}^{\circ}}$	585	Pred	
5052.989	3	19784.75	4.83	*b* ^3^H_5_*—z* ${\;}^{3}I_{5}^{{}^{\circ}}$	585	Pred	
5031.180	1	19870.51	0.38	*b* ^3^D_2_— $3_{3}^{{}^{\circ}}$?	885	New	
5025.768	3	19891.91	2.07	*c*^,3^P_1_*—v* ${\;}^{5}D_{2}^{{}^{\circ}}$	466	New	
5025.306	3	19893.74	3.69	*z* ${\;}^{3}P_{0}^{{}^{\circ}}$—*f* ^3^D_1_?		New	
5021.610	3	19908.38	8.42	*y* ${\;}^{5}F_{3}^{{}^{\circ}}$—*e* ^5^H_4_	1093	Pred	BK, U
5019.734	3	19915.82	5.81	*z* ${\;}^{3}F_{2}^{{}^{\circ}}$*—g* ^5^D_2_	966	Pred	BK, U
5019.216	1	19917.88	8.05	*d* ^3^F_2_—*u* ${\;}^{3}F_{2}^{{}^{\circ}}$	1242	Pred	
5016.494	3	19928.68	8.74	*y* ${\;}^{5}F_{3}^{{}^{\circ}}$*—g* ^5^F_2_	1089	Pred	
5015.310	3	19933.39	3.44	*z* ${\;}^{3}F_{2}^{{}^{\circ}}$—*e* ^5^P_2_	968	Pred	
5012.718	1	19943.69	3.84	*y* ${\;}^{5}F_{2}^{{}^{\circ}}$—*e* ^5^H_3_	1093	Pred	W
5011.234	2	19949.60	9.59	*y* ${\;}^{5}D_{1}^{{}^{\circ}}$—*h* ^5^D_2_	1066	Pred	W
5007.710	3	19963.64	3.53	*b* ^1^D_2_—*t* ${\;}^{3}G_{3}^{{}^{\circ}}$?		New	U
4995.406	3	20012.81	2.83	*z* ${\;}^{3}P_{1}^{{}^{\circ}}$—*f* ^5^G_2_	1113	Pred	BK
4992.814	2	20023.20	3.27	*z* ${\;}^{3}P_{1}^{{}^{\circ}}$—*g* ^5^F_1_	1110	Pred	
4991.867	3	20027.00	7.03	*y* ${\;}^{5}F_{4}^{{}^{\circ}}$—*e* ^3^G_4_	1094	Pred	U
4986.921	3	20046.86	6.94	*y* ${\;}^{5}F_{3}^{{}^{\circ}}$—*f* ^5^G_2_	1092	Pred	BK, U
4980.278	3	20073.60	3.60	*y* ${\;}^{5}F_{5}^{{}^{\circ}}$*—f* ^5^G_4_	1092	New	BK
4978.117	3	20082.31	2.36	*z* ${\;}^{3}D_{1}^{{}^{\circ}}$—*e* ^5^P_1_	986	Pred	
4942.484	3	20227.10	7.22	*y* ${\;}^{5}F_{4}^{{}^{\circ}}$—*e* ^3^H_5_	1097	New	U
4937.328	3	20248.22	8.35	*c* ^3^F_3_—*y* ${\;}^{1}F_{3}^{{}^{\circ}}$	1039	New	
4912.500	2	20350.55	0.50	*c* ^3^F_3_—*x* ${\;}^{1}F_{3}^{{}^{\circ}}$	1040	Pred b	
4908.612	0	20366.67	6.68	*a* ^3^P_0_*—x* ${\;}^{5}D_{1}^{{}^{\circ}}$	115	Pred	BK, U
4908.056	3	20368.98	9.03	*y* ${\;}^{5}D_{1}^{{}^{\circ}}$*—g* ^5^F_1_	1065	New	BK
4893.680	1	20428.82	8.75	*z* ${\;}^{3}P_{2}^{{}^{\circ}}$—*f* ^5^G_2_	1113	Pred	
4880.548	2	20483.78	3.85	*c* ^3^F_4_— $53358_{3}^{{}^{\circ}}$		New	U
4877.622	3	20496.07	6.13	*z* ${\;}^{7}P_{3}^{{}^{\circ}}$—*e* ^5^D_4_	384	Pred	
4876.194	0	20502.07	2.11	*a* ^3^D_3_—*y* ${\;}^{3}P_{2}^{{}^{\circ}}$	631	Pred	
4874.355	3	20509.81	9.83	*c* ^3^P_1_—*x* ${\;}^{3}D_{2}^{{}^{\circ}}$	467	Pred	
4873.758	2	20512.32	2.41	*a* ^3^D_2_—*w* ${\;}^{3}D_{2}^{{}^{\circ}}$	633	Pred	
4870.081	3	20527.81	7.95	*z* ${\;}^{3}D_{2}^{{}^{\circ}}$—*g* ^5^D_1_	985	Pred	
4869.476	3	20530.36	0.47	*a* ^1^D_2_—*v* ${\;}^{3}D_{3}^{{}^{\circ}}$	751	Pred	
4868.382	3	20534.97	5.01	*a* ^3^F_3_—*y* ${\;}^{5}D_{4}^{{}^{\circ}}$	38	Pred	
4867.563	1	20538.42	8.57	*a* ^3^F_2_—*y* ${\;}^{5}D_{3}^{{}^{\circ}}$	38	Pred	U
4860.988	3	20566.20	6.27	*z* ${\;}^{5}F_{3}^{{}^{\circ}}$*—e* ^3^F_4_	688	SS	U, ZZ
4858.274	3	20577.69	$\left\{ \;\begin{array}{l} 7.74\\ 7.84 \end{array}\right.$	*y* ${\;}^{5}F_{2}^{{}^{\circ}}$*—f* ^3^F_3_	1098	Pred	} BK
				*y* ${\;}^{5}D_{2}^{{}^{\circ}}$*—e* ^3^G_3_	1069	New	
4849.655	2	20614.26	4.22	*a* ^1^H_5_—*y* ${\;}^{3}H_{6}^{{}^{\circ}}$	793	Pred	
4847.699	0	20622.58	2.55	*a* ^1^D_2_— $3_{3}^{{}^{\circ}}$		New	
4838.086	2	20663.56	3.55	*a* ^3^D_3_*—u* ${\;}^{5}D_{2}^{{}^{\circ}}$	630	Pred	
4837.662	0	20665.37	5.42	*d* ^3^F_3_—*t* ${\;}^{3}F_{3}^{{}^{\circ}}$	1243	Pred	
4822.684	1*n*	20729.55	9.65	*a* ^3^D_1_—*w* ${\;}^{3}D_{2}^{{}^{\circ}}$	633	Pred	Z
4821.028	0	20736.67	6.76	*c* ^3^F_4_— $53610_{4}^{{}^{\circ}}$		New	
4815.238	3	20761.60	1.71	*a* ^1^P_1_—*x* ${\;}^{3}P_{2}^{{}^{\circ}}$	720	Pred	
4805.529	0*n*	20803.55	3.59	*y* ${\;}^{5}P_{1}^{{}^{\circ}}$—4_2_	1207	New	
4802.503	2*n*	20816.66	6.57	*y* ${\;}^{5}P_{2}^{{}^{\circ}}$—*i* ^5^D_2_	1206	Pred	
4794.353	2	20852.04	2.05	*a* ^3^P_1_—*x* ${\;}^{5}D_{1}^{{}^{\circ}}$	115	Pred	BK, U
4793.951	2	20853.79	3.77	*a* ^1^G_4_—*y* ${\;}^{3}G_{4}^{{}^{\circ}}$	512	(a)	W
4792.537	2*n*	20859.94	0.03	*y* ${\;}^{5}F_{5}^{{}^{\circ}}$—*e* ^3^H_4_	1097	New	
4790.764	1	20867.66	7.75	*a* ^3^D_3_—*x* ${\;}^{3}F_{3}^{{}^{\circ}}$	632	Pred	BK, U
4790.542	0*n*	20868.63	8.58	*y* ${\;}^{5}D_{3}^{{}^{\circ}}$—*f* ^5^G_2_	1068	Pred	
4782.789	1*n*	20902.46	2.48	*b* ^3^H_6_—*z* ${\;}^{3}H_{5}^{{}^{\circ}}$	588	Pred	
4780.789	2	20911.20	1.11	*a* ^3^D_3_*—w* ${\;}^{3}D_{2}^{{}^{\circ}}$	633	Pred	
4773.496	0	20943.15	3.08	*b* ^3^G_3_*—x* ${\;}^{3}D_{2}^{{}^{\circ}}$	408	Pred b	
4760.050	1	21002.31	2.23	*z* ${\;}^{7}P_{2}^{{}^{\circ}}$—*e* ^5^D_1_	384	Pred	U
4758.689	1	21008.32	8.23	*c* ^3^F_4_— $53882_{4}^{{}^{\circ}}$		New	
4749.580	1	21048.61	8.55	*a* ^3^F_2_—*y* ${\;}^{5}D_{1}^{{}^{\circ}}$	38	New	
4744.615	1*n*	21070.63	0.55	*a* ^5^F_2_—*z* ${\;}^{5}P_{3}^{{}^{\circ}}$	17	Pred	BK, U
4742.920	1	21078.16	8.12	*y* ${\;}^{5}D_{2}^{{}^{\circ}}$*—e* ^3^P_2_	1072	Pred	
4718.410	1	21187.65	7.57	*c* ^3^F_3_*—t* ${\;}^{3}G_{3}^{{}^{\circ}}$	1042	New	
4716.816	0*n*	21194.81	4.69	*a* ^3^D_3_— $1_{2}^{{}^{\circ}}$	634	Pred	
4702.926	0	21257.41	7.44	*b* ^3^H_6_*—w* ${\;}^{5}G_{6}^{{}^{\circ}}$		New	
4690.367	1	21314.33	4.31	*a* ^5^F_1_—*z* ${\;}^{5}P_{2}^{{}^{\circ}}$	17	Pred	BK, U
4685.036	3	21338.58	8.62	*b* ^3^P_1_—*w* ${\;}^{5}F_{2}^{{}^{\circ}}$	347	Pred	
4677.572	2*n*	21372.63	2.58	*y* ${\;}^{5}D_{3}^{{}^{\circ}}$*—e* ^3^P_2_	1072	Pred	
4674.651	2	21385.98	6.01	*a* ^3^F_3_—*z* ${\;}^{3}P_{2}^{{}^{\circ}}$	40	Pred	U
4665.522	0	21427.83	7.68	*c* ^3^F_4_— $13_{4}^{{}^{\circ}}$?	1044	Pred	
4653.446	1	21483.44	3.53	*b* ^3^H_5_—*x* ${\;}^{3}G_{5}^{{}^{\circ}}$	591	New	BK
4642.624	1	21533.51	3.72	*z* ${\;}^{5}P_{3}^{{}^{\circ}}$—*e* ^3^F_2_?	688	Pred	
4636.072	1	21561.16	1.23	*a* ^1^G_4_*—z* ${\;}^{3}I_{5}^{{}^{\circ}}$	513	Pred	
4634.170	1	21572.80	2.82	*b* ^3^P_2_—*w* ${\;}^{5}D_{1}^{{}^{\circ}}$	346	New	
4628.696	0	21598.31	8.34	*z* ${\;}^{5}P_{1}^{{}^{\circ}}$*—e* ^7^F_2_	819	Pred	
4627.532	2	21603.74	3.69	*b* ^3^H_4_—*w* ${\;}^{3}G_{5}^{{}^{\circ}}$	593	New	
4605.070	0	21709.12	9.00	*b* ^3^P_0_*—v* ${\;}^{5}D_{1}^{{}^{\circ}}$	348	Pred	
4604.850	0	21710.15	0.15	*a* ^1^I_6_—*x* ${\;}^{3}H_{6}^{{}^{\circ}}$	846	Pred	
4598.728	1	21739.05	9.01	*z* ${\;}^{5}P_{2}^{{}^{\circ}}$*—e* ^7^F_1_	819	Pred	
4591.502	2*n*	21773.26	3.09	*a* ^3^G_3_*—w* ${\;}^{5}F_{4}^{{}^{\circ}}$?		New	U
4583.726	2	21810.20	0.27	*c* ^3^P_0_*—y* ${\;}^{3}P_{1}^{{}^{\circ}}$	472	Pred	
4572.849	2	21862.08	2, 06	*z* ${\;}^{5}P_{2}^{{}^{\circ}}$—*e* ^7^F_2_	819	(a)	U, W
4571.448	2	21868.78	8.83	*z* ${\;}^{7}F_{2}^{{}^{\circ}}$*—e* ^5^D_3_	319	Pred	U
4561.426	2	21916.83	6.78	*a* ^3^G_3_—*w* ${\;}^{5}F_{3}^{{}^{\circ}}$		New	U
4546.477	1	21988.89	8.96	*c* ^3^F_2_—*w* ${\;}^{1}D_{2}^{{}^{\circ}}$	1047	Pred	
4541.319	2	22013.86	3.87	*a* ^3^D_3_*—v* ${\;}^{5}F_{2}^{{}^{\circ}}$	640	New	BK, U
4540.651	1	22017.10	7.07	*b* ^3^G_4_*—z* ${\;}^{3}I_{5}^{{}^{\circ}}$	411	New	U
4533.953	1	22049.63	9.59	*b* ^3^G_5_—*x* ${\;}^{5}G_{4}^{{}^{\circ}}$	410	New	
4520.240	2	22116.52	6.52	*c* ^3^P_1_*—u* ${\;}^{5}D_{2}^{{}^{\circ}}$	471	(a,c)	BK, Z
4518.583	2	22124.63	4.67	*a* ^5^P_1_—*y* ${\;}^{7}P_{2}^{{}^{\circ}}$	69	Pred	
4516.265	1	22135.98	5.98	*z* ${\;}^{5}P_{3}^{{}^{\circ}}$*—e* ^7^F_4_	819	Pred	U
4515.146	2	22141.47	1.37	*z* ${\;}^{7}F_{2}^{{}^{\circ}}$*—e* ^5^D_2_	319	SS	U, ZZ
4513.713	1	22148.50	8.48	*b ^3^*F_3_*—y* ${\;}^{5}G_{4}^{{}^{\circ}}$	213	Pred b	U
4507.232	1	22180.34	0.48	*c* ^3^P_0_*—w* ${\;}^{3}D_{1}^{{}^{\circ}}$	474	New c	
4487.748	1	22276.64	6.68	*b* ^3^H_6_—*z* ${\;}^{1}H_{5}^{{}^{\circ}}$	594	Pred	U
4483.771	1	22296.40	6.36	*b* ^3^D_3_—*u* ${\;}^{3}G_{4}^{{}^{\circ}}$	898	Pred	U
4474.722	1	22341.49	1.51	*c* ^3^F_3_—*w* ${\;}^{1}D_{2}^{{}^{\circ}}$	1047	New	
4473.004	0	22350.07	0.24	*z* ${\;}^{7}F_{1}^{{}^{\circ}}$*—e* ^5^D_0_?	319	New	
4463.147	2	22399.43	$\left\{ \begin{array}{l} 9.46\\ 9.38 \end{array}\right.$	*c* ^3^P_1_*—u* ${\;}^{5}D_{0}^{{}^{\circ}}$ *b* ^3^D_2_— $7_{2}^{{}^{\circ}}$	471 901	Pred Pred b	} U
4453.325	2	22448.83	9.03	*z* ${\;}^{5}D_{1}^{{}^{\circ}}$*—e* ^3^F_2_	555	New	
4452.616	1*n*	22452.40	2.42	*z* ${\;}^{3}F_{3}^{{}^{\circ}}$*—g* ^5^F_2_	969	Pred	U
4450.765	2	22461.74	1.75	*z* ${\;}^{3}F_{4}^{{}^{\circ}}$*—f* ^5^G_4_	972	Pred	U
4443.885	1	22496.52	6.37	*b* ^3^F_4_—*y* ${\;}^{5}G_{3}^{{}^{\circ}}$?	213	New	
4437.695	1	22527.90	7.90	*a* ^3^G_5_—*w* ${\;}^{5}F_{5}^{{}^{\circ}}$		New	
4428.554	2	22574.40	4.34	*z* ${\;}^{3}F_{3}^{{}^{\circ}}$—*e* ^3^G_3_	973	Pred	U
4419.790	0	22619.16	9.22	*a* ^3^D_2_*—w* ${\;}^{3}F_{3}^{{}^{\circ}}$	644	Pred	
4419.076	2*n*	22622.81	2.64	*z* ${\;}^{3}G_{5}^{{}^{\circ}}$*—g* ^5^G_6_?	1170	New	
4417.334	1*n*	22631.73	1.89	*z* ${\;}^{5}D_{4}^{{}^{\circ}}$*—e* ^3^F_3_	555	New	
4407.230	3	22683.62	3.62	*z* ${\;}^{5}P_{3}^{{}^{\circ}}$*—e* ^3^D_2_	827	New	U
4393.014	0	22757.02	$\left\{ \;\begin{array}{l} 6.85\\ 6.97 \end{array}\right.$	*a* ^1^D_2_—*t* ${\;}^{5}D_{3}^{{}^{\circ}}$?	473	New	
				*c* ^3^P_2_—*x* ${\;}^{3}F_{3}^{{}^{\circ}}$		Pred	
4391.865	1	22762.98	2.95	*z* ${\;}^{3}D_{2}^{{}^{\circ}}$—*f* ^3^D_1_	992	Pred	
4370.982	1	22871.73	1.68	*a* ^5^P_3_—*y* ${\;}^{7}P_{4}^{{}^{\circ}}$	69	Pred^b^	
4350.972	1	22976.91	6.81	*b* ^3^H_4_—*y* ${\;}^{3}H_{5}^{{}^{\circ}}$	597	New	
4341.802	0*n*	23025.44	5.46	*a* ^1^D_2_—*t* ${\;}^{5}D_{2}^{{}^{\circ}}$		New	
4341.248	1	23028.38	8.48	*z* ${\;}^{5}F_{3}^{{}^{\circ}}$—*f* ^5^D_4_	691	Pred	U
4340.490	1	23032.40	$\left\{ \;\begin{array}{l} 2.30\\ 2.43 \end{array}\right.$	*z* ${\;}^{5}F_{1}^{{}^{\circ}}$—*f* ^5^D_2_ *a* ^3^G_3_—*x* ${\;}^{3}D_{2}^{{}^{\circ}}$	691 272	} (a)	U, W
4330.812	1	23083.87	3.92	*c* ^3^P_2_— $1_{2}^{{}^{\circ}}$	475	Pred	U
4319.433	1*n*	23144.68	4.62	*b* ^3^F_2_—*w* ${\;}^{5}D_{2}^{{}^{\circ}}$	214	Pred	BK, U
4310.363	1	23193.38	3.34	*z* ${\;}^{3}D_{2}^{{}^{\circ}}$—*e* ^3^P_2_	994	Pred	U
4305.128	1	23221.58	1.57	*a* ^3^G_4_*—x* ${\;}^{3}D_{3}^{{}^{\circ}}$	272	Pred^b^	
4304.878	0*n*	23222.93	$\left\{ \;\begin{array}{l} 2.98\\ 2.97 \end{array}\right.$	*a* ^1^D_2_*—u* ${\;}^{5}F_{2}^{{}^{\circ}}$ *b* ^3^H_4_*—v* ${\;}^{3}G_{3}^{{}^{\circ}}$	756 598	Pred^b^ Pred^b^	} BK, U
4304.105	0	23226.78	6.88	*a* ^3^D_2_*—v* ${\;}^{3}G_{3}^{{}^{\circ}}$	647	Pred b	
4300.205	1*n*	23248.16	8.18	*z* ${\;}^{3}F_{4}^{{}^{\circ}}$*—e* ^3^H_4_	975	Pred	
4292.136	1	23291.87	$\left\{ \begin{array}{l} 1.72\\ 1.92 \end{array}\right.$	*b* ^3^F_3_—*w* ${\;}^{5}F_{3}^{{}^{\circ}}$	215	New	} BK, U
				*a* ^5^P_3_—*x* ${\;}^{5}F_{3}^{{}^{\circ}}$	70	Pred	
4289.924	2	23303.88	3.96	*z* ${\;}^{5}F_{3}^{{}^{\circ}}$*—f* ^5^D_2_	691	New	U
4286.872	1*n*	23320.47	$\left\{ \;\begin{array}{l} 0.55\\ 0.66 \end{array}\right.$	*z* ${\;}^{5}F_{2}^{{}^{\circ}}$*—f* ^5^D_1_ *a* ^3^H_4_—*z* ${\;}^{5}H_{4}^{{}^{\circ}}$	691 172	New New	} V, Z
4283.384	1*n*	23339.46	9.40	*b* ^3^F_2_*—w* ${\;}^{5}F_{1}^{{}^{\circ}}$	215	Pred	BK, U
4277.389	1	23372.17	2.16	*b* ^3^F_2_*—*w ${\;}^{5}D_{1}^{{}^{\circ}}$	214	Pred	
4275.688	2*n*	23381.47	1.41	*b* ^3^F_4_—*w* ${\;}^{5}F_{4}^{{}^{\circ}}$	215	(a)	U
4273.335	1*n*	23394.34	4.19	*a* ^3^H_6_—*y* ${\;}^{5}G_{6}^{{}^{\circ}}$?	171	New c	U, W
4259.332	0	23471.25	1.22	*b* ^3^G_4_—*w* ${\;}^{5}G_{4}^{{}^{\circ}}$	416	Pred	U
4256.289	1	23488.03	7.91	*a* ^3^H_5_—*z* ${\;}^{5}H_{4}^{{}^{\circ}}$	172	Pred	
4253.933	1	23501.04	1.06	*b* ^3^D_2_— $8_{1}^{{}^{\circ}}$	905	Pred	Z
4246.540	2	23541.96	1.68	*z* ${\;}^{5}F_{1}^{{}^{\circ}}$—*e* ^7^F_1_?	689	SS	U, ZZ
4243.560	2	23558.49	8.57	*b* ^1^G_4_— $53358_{3}^{{}^{\circ}}$		New	U
4239.375	3	23581.74	1.82	*b* ^3^D_3_*—s* ${\;}^{3}D_{3}^{{}^{\circ}}$	907	Pred	U
4237.675	1	23591.20	1.24	*b* ^3^G_3_*—v* ${\;}^{5}F_{4}^{{}^{\circ}}$	418	Pred	U, ZZ
4228.705	1*n*	23641.24	1.22	*z* ${\;}^{5}F_{4}^{{}^{\circ}}$—*f* ^7^D_4_	690	SS	
4223.722	2	23669.13	9.12	*b* ^3^G_5_—*z* ${\;}^{1}G_{4}^{{}^{\circ}}$	417	Pred	
4220.034	1	23689.82	9.74	*z* ${\;}^{3}D_{2}^{{}^{\circ}}$*—e* ^3^P_1_	994	Pred	U
4197.088	2	23819.33	9.30	*a* ^5^F_2_*—z* ${\;}^{3}F_{3}^{{}^{\circ}}$	18	Pred	U
4194.479	1	23834.15	4.07	*a* ^3^G_4_—*x* ${\;}^{5}G_{4}^{{}^{\circ}}$	274	Pred	BK, U
4188.729	2*n*	23866.86	7.01	*z* ${\;}^{3}P_{2}^{{}^{\circ}}$*—i* ^5^D_3_	1116	New	U
4181.210	1*n*	23909.78	9.89	*b* ^3^D_1_—*z* ${\;}^{1}P_{1}^{{}^{\circ}}$	908	Pred	U
4180.404	1	23914.39	4.36	*a* ^3^G_4_—*x* ${\;}^{5}G_{3}^{{}^{\circ}}$	274	Pred	BK, U
*4167.960*	1	23985.79	5.67	*b* ^3^D_3_— $53358_{3}^{{}^{\circ}}$?		New	V
4149.759	3	24090.99	1.00	*a* ^5^D_3_*—z* ${\;}^{7}P_{2}^{{}^{\circ}}$	3	SS	U, ZZ
4140.240	1	24146.38	6.39	*b* ^3^G_5_*—v* ${\;}^{5}F_{4}^{{}^{\circ}}$	418	Pred^b^	U
4137.980	1	24159.57	9.63	*z* ${\;}^{7}F_{5}^{{}^{\circ}}$*—e* ^5^F_5_	320	Pred	
4137.456	1	24162.63	2.85	*y* ${\;}^{5}F_{2}^{{}^{\circ}}$—*g* ^5^G_3_	1103	Pred	U
4134.202	1*N*	24181.64	1.72	*b* ^3^F_2_—*x* ${\;}^{3}D_{3}^{{}^{\circ}}$	217	Pred	U
4129.474	1	24209.33	9.44	*z* ${\;}^{5}F_{3}^{{}^{\circ}}$*—f* ^5^F_3_	695	Pred	
4124.490	1	24238.58	8.58	*b* ^3^D_3_— $53610_{4}^{{}^{\circ}}$		New	U
4112.094	1	24311.65	1.72	*a* ^1^D_2_—*v* ${\;}^{3}P_{2}^{{}^{\circ}}$	766	Pred	U
4108.129	1	24335.11	5.13	*z* ${\;}^{5}D_{3}^{{}^{\circ}}$*—e* ^7^P_4_	559	Pred	U
4104.460	1	24356.87	6.89	*b* ^3^G_4_—*w* ${\;}^{3}G_{3}^{{}^{\circ}}$	422	Pred	U
4103.620	2	24361.85	1.86	*a* ^3^D_3_—*z* ${\;}^{1}F_{3}^{{}^{\circ}}$	650	Pred	U
4095.642	1	24409.31	9.40	*a* ^1^I_6_—*y* ${\;}^{1}H_{5}^{{}^{\circ}}$	851	Pred	
4095.252	1*N*	24411.63	1.57	*y* ${\;}^{5}D_{2}^{{}^{\circ}}$—4_2_	1075	Pred	
*4092.287*	1	24429.32	9.34	*b* ^3^D_1_— $53749_{2}^{{}^{\circ}}$		New	J
4090.326	1	24441.03	1.00	*a* ^3^F_2_—*y* ${\;}^{5}P_{1}^{{}^{\circ}}$	44	Pred	BK, U
4079.214	3*N*	24507.61	7.85	*z* ${\;}^{5}F_{2}^{{}^{\circ}}$—*e* ^5^P_2_	700	Pred	BK, U
*4078.822*	1	24509.96	0.05	*b* ^3^D_1_— $53882_{4}^{{}^{\circ}}$		New	BK
4070.422	0	24560.54	0.39	*a* ^1^G_4_—*v* ${\;}^{3}D_{3}^{{}^{\circ}}$?	525	Pred^b^	
4070.010	0	24563.03	3.03	*z* ${\;}^{7}F_{2}^{{}^{\circ}}$—*e* ^5^F_3_	320	New	BK
4057.654	1	24637.82	7.82	*a* ^1^P_1_—*t* ${\;}^{3}D_{1}^{{}^{\circ}}$	729	Pred	U
4036.370	1	24767.74	7.78	*a* ^3^G_3_—*w* ${\;}^{3}D_{3}^{{}^{\circ}}$	279	Pred	BK, U
4031.727	2	24796.26	6.28	*b* ^3^G_3_—*v* ${\;}^{3}D_{3}^{{}^{\circ}}$	427	Pred	U
4022.212	1*n*	24854.92	4.93	*b* ^3^P_2_—*w* ${\;}^{5}G_{3}^{{}^{\circ}}$	360	New	BK, U, W
4001.212	1*N*	24985.36	5.54	*b* ^3^D_3_— $54357_{3}^{{}^{\circ}}$		New	BK, U
3998.743	0	25000.79	0.62	*b* ^3^D_2_— $54357_{3}^{{}^{\circ}}$		New	
3996.779	1	25013.08	3.04	*y* ${\;}^{5}D_{3}^{{}^{\circ}}$—*g* ^5^G_4_	1074	Pred	U
3996.261	1*n*	25016.32	$\left\{ \;\begin{array}{l} 6.36\\ 6.23 \end{array}\right.$	*z* ${\;}^{5}D_{0}^{{}^{\circ}}$—*e* ^7^G_1_	561	Pred	} U
				*b* ^3^G_4_—*v* ${\;}^{3}D_{3}^{{}^{\circ}}$	427	Pred	
3992.634	1	25039.04	9.01	*b* ^3^F_3_—*x* ${\;}^{5}G_{3}^{{}^{\circ}}$	219	Pred	BK, U
3989.008	1*N*	25061.81	1.93	*a* ^1^H_5_— $53882_{4}^{{}^{\circ}}$		New	Z
3984.930	1	25087.45	7.47	*z* ${\;}^{5}D_{1}^{{}^{\circ}}$—*e* ^7^G_1_	561	Pred	Z
3984.446	1	25090.50	0.46	*b* ^3^F_3_—*x* ${\;}^{5}G_{2}^{{}^{\circ}}$	219	Pred	BK
3980.008	1	25118.47	8.56	*b* ^3^G_3_— $49457_{4}^{{}^{\circ}}$?		New	U
3970.994	1	25175.49	5.54	*y* ${\;}^{5}D_{4}^{{}^{\circ}}$—*g* ^5^G_5_	1074	Pred	U
3963.438	1	25223.48	3.58	*a* ^3^D_1_—*t* ${\;}^{5}D_{2}^{{}^{\circ}}$	654	Pred^b^	U
3962.635	0*N*	25228.60	8.49	*b* ^3^D_3_—*t* ${\;}^{3}G_{3}^{{}^{\circ}}$	913	Pred	
3953.512	2	25286.81	6.93	*a* ^1^D_2_— $10_{3}^{{}^{\circ}}$	770	Pred^b^	U
3951.638	1*N*	25298.80	8.83	*b* ^3^P_0_—*v* ${\;}^{5}F_{1}^{{}^{\circ}}$	362	New	U
3948.458	1	25319.18	9.06	*z* ${\;}^{5}D_{4}^{{}^{\circ}}$—*e* ^5^G_3_	560	Pred	BK
3930.876	0*N*	25432.42	2.46	*a* ^3^H_4_—*x* ${\;}^{3}D_{3}^{{}^{\circ}}$?		New	TJ
3922.100	1*N*	25489.33	$\left\{ \;\begin{array}{l} 9.51\\ 9.44 \end{array}\right.$	*z* ${\;}^{7}D_{1}^{{}^{\circ}}$—*e* ^5^D_1_	153	Pred	} BK, U
				*z* ${\;}^{5}D_{0}^{{}^{\circ}}$—*e* ^3^D_1_	564	Pred	
3908.691	1*N*	25576.77	6.84	*z* ${\;}^{7}D_{3}^{{}^{\circ}}$—*e* ^5^D_2_	153	Pred	BK
3892.302	1	25684.46	4.48	*a* ^1^D_2_— $54289_{3}^{{}^{\circ}}$		New	U
3889.284	1*N*	25704.39	4.46	*a* ^3^G_5_—*w* ${\;}^{5}G_{5}^{{}^{\circ}}$	280	New	U
3847.226	1*N*	25985.38	5.44	*b* ^3^H_4_—*w* ${\;}^{3}H_{5}^{{}^{\circ}}$	607	New	
3847.077	1*N*	25986.39	6.28	*z* ${\;}^{5}F_{2}^{{}^{\circ}}$—*h* ^5^D_3_	702	New	BK, U
3842.901	2	26014.63	4.69	*b* ^3^F_3_—*x* ${\;}^{3}F_{4}^{{}^{\circ}}$	222	Pred	BK, Z
3819.497	2*N*	26174.03	4.02	*z* ${\;}^{5}F_{3}^{{}^{\circ}}$—*f* ^5^P_2_	703	Pred	Z
3816.908	1	26191.78	1.74	*z* ${\;}^{7}P_{2}^{{}^{\circ}}$—*f* ^5^D_2_	387	Pred	U
*3814.785*	1	26206.36	6.39	*a* ^1^P_1_— $53749_{2}^{{}^{\circ}}$		New	Z
3811.808	0	26226.83	6.88	*z* ${\;}^{5}F_{4}^{{}^{\circ}}$—*g* ^5^F_4_	701	Pred	BK, U
3803.220	1*N*	26286.05	5.91	*a* ^3^P_2_—*v* ${\;}^{5}D_{2}^{{}^{\circ}}$	122	Pred	U
3801.337	1	26299.07	8.89	*b* ^1^G_4_—*s* ${\;}^{3}G_{3}^{{}^{\circ}}$	948	New	Z
3789.808	1*N*	26379.07	9.04	*z* ${\;}^{5}F_{4}^{{}^{\circ}}$—*h* ^5^D_3_	702	Pred	Z
3789.292	0	26382.66	2.57	*a* ^3^P_2_—*v* ${\;}^{5}D_{1}^{{}^{\circ}}$	122	New	
3784.127	3*n*	26418.67	8.86	*b* ^3^D_3_—*w* ${\;}^{1}F_{3}^{{}^{\circ}}$	917	New	U
3771.473	2	26507.31	7.13	*b* ^3^H_6_—*w* ${\;}^{3}H_{5}^{{}^{\circ}}$	607	Pred	BK, Z
3769.766	0	26519.31	9.15	*z* ${\;}^{5}F_{5}^{{}^{\circ}}$—*g* ^5^F_4_	701	New	
3759.597	1*n*	26591.04	1.16	*z* ${\;}^{5}F_{1}^{{}^{\circ}}$—*g* ^5^F_2_	701	New	U
3751.087	2	26651.36	1.42	*a* ^5^P_2_—*w* ${\;}^{5}F_{1}^{{}^{\circ}}$	74	Pred^d^	Z
3743.778	2	26703.39	3.40	*a* ^3^G_4_—*y* ${\;}^{1}G_{4}^{{}^{\circ}}$	290	SS	Z, ZZ
3742.151	2*n*	26715.00	5.08	*z* ${\;}^{3}F_{3}^{{}^{\circ}}$—*g* ^5^G_4_	978	Pred	U
3741.486	1*n*	26719.75	9.80	*z* ${\;}^{5}F_{1}^{{}^{\circ}}$—*g* ^5^F_1_	701	New	BK, Z
*3739.527*	3	26733.75	3.80	*a* ^3^D_3_— $53358_{3}^{{}^{\circ}}$		New	J
3707.578	1*n*	26964.11	4.23	*z* ${\;}^{3}F_{4}^{{}^{\circ}}$—*g* ^5^G_5_	978	New	Z
3707.458	2	26964.99	4.96	*b* ^3^F_4_—*v* ${\;}^{5}F_{5}^{{}^{\circ}}$	229	New	Z
3707.335	1	26965.88	5.85	*b* ^3^G_3_—*v* ${\;}^{3}F_{4}^{{}^{\circ}}$	437	New	U
3699.004	0	27026.61	6.50	*b* ^3^G_3_—*v* ${\;}^{3}F_{3}^{{}^{\circ}}$	437	New	
3698.148	1*n*	27032.87	2.84	*z* ${\;}^{7}P_{2}^{{}^{\circ}}$—*e* ^7^G_2_	390	New	W, Z
3698.018	1	27033.82	3.77	*a* ^5^P_2_—*v* ${\;}^{5}D_{1}^{{}^{\circ}}$	75	Pred	U
3696.548	1*n*	27044.57	4.45	*a* ^1^G_4_—*u* ${\;}^{5}F_{3}^{{}^{\circ}}$	530	New	
3691.180	0	27083.90	3.95	*b* ^3^F_2_—*v* ${\;}^{5}F_{3}^{{}^{\circ}}$	229	Pred	Z
3689.010	1	27099.83	9.81	*a* ^3^H_5_—*u* ${\;}^{5}D_{4}^{{}^{\circ}}$	178	Pred^b^	U
3688.198	1	27105.80	5.87	*b* ^3^H_4_— $53734_{3}^{{}^{\circ}}$		New	U
3684.552	1	27132.62	2.50	*a* ^3^D_3_— $53358_{3}^{{}^{\circ}}$		New	
*3681.227*	2	27157.12	7.10	*b* ^3^H_4_— $53785_{3}^{{}^{\circ}}$		New	Z
3677.503	2	27184.62	4.64	*a* ^3^D_2_—*v* ${\;}^{3}P_{1}^{{}^{\circ}}$	666	New d	V, Z
3675.694	1*n*	27198.00	8.12	*z* ${\;}^{7}P_{2}^{{}^{\circ}}$—*f* ^5^F_2_	391	New	Z
3675.434	1	27199.93	9.88	*b* ^3^F_2_—*v* ${\;}^{5}F_{2}^{{}^{\circ}}$	229	Pred	U
3671.689	2	27227.67	7.64	*a* ^3^G_3_—*z* ${\;}^{1}D_{2}^{{}^{\circ}}$		New	Z
3666.846	1*n*	27263.63	3.65	*z* ${\;}^{7}P_{2}^{{}^{\circ}}$—*g* ^5^D_3_	393	Pred	
3666.574	0	27265.65	5.69	*b ^3^*F_2_—*x* ${\;}^{3}P_{2}^{{}^{\circ}}$	235	New	U
3660.402	2	27311.62	1.60	*b* ^3^F_2_—*v* ${\;}^{5}F_{1}^{{}^{\circ}}$	229	Pred	
*3656.227*	2	27342.81	2.90	*a* ^3^D_1_— $53749_{2}^{{}^{\circ}}$		New	V
3655.354	1	27349.34	9.40	*a* ^3^P_1_—*y* ${\;}^{3}P_{1}^{{}^{\circ}}$	131	Pred	U
3653.352	1*n*	27364.33	$\left\{ \;\begin{array}{l} 4.38\\ 4.37 \end{array}\right.$	*b* ^3^F_3_—*v* ${\;}^{5}F_{2}^{{}^{\circ}}$	229	Pred	} U
				*z* ${\;}^{7}F_{3}^{{}^{\circ}}$—*e* ^7^P_4_	324	Pred	
3652.256	1*n*	27372.54	2.53	*c* ^3^P_2_—*y* ${\;}^{1}D_{2}^{{}^{\circ}}$	494	Pred	U
3650.554	1*n*	27385.30	5.41	*a* ^3^D_3_— $53610_{4}^{{}^{\circ}}$		New	W, Z
3643.812	2*n*	27435.97	$\left\{ \;\begin{array}{l} 5.91\\ 6.10 \end{array}\right.$	*a* ^3^F_2_—*x* ${\;}^{5}D_{1}^{{}^{\circ}}$	46	} SS	Z, ZZ
				*a* ^3^D_3_—*y* ${\;}^{1}F_{3}^{{}^{\circ}}$	670		
3641.454	2	27453.73	3.82	*z* ${\;}^{7}F_{1}^{{}^{\circ}}$—*f* ^5^D_2_	323	Pred	
3637.044	2	27487.02	7.00	*b* ^3^G_3_—*u* ${\;}^{3}G_{3}^{{}^{\circ}}$	438	Pred	BK
3636.496	2*n*	27491.16	$\left\{ \;\begin{array}{l} 1.25\\ 1.13 \end{array}\right.$	*z* ${\;}^{5}D_{2}^{{}^{\circ}}$—*g* ^5^F_3_	568	Pred	} BK, Z
				*a* ^3^F_3_—*y* ${\;}^{7}P_{2}^{{}^{\circ}}$	47	Pred	
3633.087	3*n*	27516.96	7.13	*z* ${\;}^{7}P_{4}^{{}^{\circ}}$—*e* ^7^G_5_	390	SS	Z, ZZ
3628.806	2	27549.42	9.37	*b* ^3^G_4_—*u* ${\;}^{3}G_{4}^{{}^{\circ}}$	438	Pred	Z
3624.056	1	27585.53	5.55	*z* ${\;}^{5}D_{2}^{{}^{\circ}}$—*f* ^5^P_1_	570	Pred	Z
3620.880	2*n*	27609.72	$\left\{ \;\begin{array}{l} 9.76\\ 9.82 \end{array}\right.$	*z* ${\;}^{7}F_{0}^{{}^{\circ}}$—*f* ^5^D_1_	323	Pred	} BK, U
				*b* ^3^H_4_—*t* ${\;}^{3}G_{4}^{{}^{\circ}}$	611	Pred	
3619.628	1	27619.27	9.03	*a* ^3^P_1_—*u* ${\;}^{5}D_{0}^{{}^{\circ}}$	130	Pred	BK, U
3618.285	3	27629.52	9.44	*z* ${\;}^{7}F_{5}^{{}^{\circ}}$—*e* ^7^P_4_	324	Pred	BK, Z
3618.160	2	27630.48	0.34	*b* ^3^G_3_—*u* ${\;}^{3}D_{3}^{{}^{\circ}}$		New	
3617.946	3	27632.11	1.95	*a* ^3^H_4_—*w* ${\;}^{5}G_{5}^{{}^{\circ}}$	181	Pred	U
3616.162	3	27645.74	5.88	*z* ${\;}^{5}D_{4}^{{}^{\circ}}$—*h* ^5^D_3_	569	SS	Z, ZZ
3615.959	1	27647.30	7.38	*a* ^3^D_2_—*t* ${\;}^{5}P_{1}^{{}^{\circ}}$		New	BK, U, W
3615.024	0	27654.45	4.62	*z* ${\;}^{7}D_{5}^{{}^{\circ}}$—*e* ^5^F_5_	154	Pred	
*3614.711*	3	27656.84	6.88	*a* ^3^D_3_— $53882_{4}^{{}^{\circ}}$		New	BK
*3614.109*	1	27661.45	1.45	*b* ^3^H_4_— $54289_{3}^{{}^{\circ}}$		New	BK
3613.950	0	27662.66	2.69	*b* ^3^H_5_— $12_{5}^{{}^{\circ}}$	612	Pred	
3613.711	1	27664.49	4.49	*a* ^3^H_4_—*z* ${\;}^{1}G_{4}^{{}^{\circ}}$		New	
*3613.612*	2	27665.25	5.36	*a* ^3^D_2_— $54289_{3}^{{}^{\circ}}$		New	Z
3613.459	3	27666.42	6.51	*a* ^3^D_3_— $10_{3}^{{}^{\circ}}$	672	Pred	BK, Z
3612.510	3	27673.69	3.72	*b* ^3^H_4_— $13_{4}^{{}^{\circ}}$	613a	Pred	Z
3610.410	1	27689.79	9.93	*c* ^3^F_4_— $60564_{3}^{{}^{\circ}}$?		New	
3609.486	2	27696.88	7.10	*z* ${\;}^{7}F_{3}^{{}^{\circ}}$—*f* ^7^D_4_	322	Pred	
3606.504	3	27719.78	9.60	*a* ^3^P_1_—*w* ${\;}^{3}D_{1}^{{}^{\circ}}$	133	Pred	
3606.363	2	27720.86	0.78	*b* ^3^F_4_—*w* ${\;}^{3}G_{4}^{{}^{\circ}}$	233	Pred	U
*3605.206*	1	27729.76	9.76	*b* ^3^H_4_— $54357_{3}^{{}^{\circ}}$		New	BK
3604.701	2	27733.64	3.67	*a* ^3^D_2_— $54357_{3}^{{}^{\circ}}$		New	BK
3603.673	2	27741.55	$\left\{ \;\begin{array}{l} 1.49\\ 1.59 \end{array}\right.$	*b* ^3^F_4_—*z* ${\;}^{1}H_{5}^{{}^{\circ}}$		New	} U
				*a* ^3^G_5_— $49457_{4}^{{}^{\circ}}$		New	
3602.774	1	27748.47	8.53	*b* ^3^P_2_—*z* ${\;}^{1}F_{3}^{{}^{\circ}}$	370	Pred^b^	U
3601.429	1*n*	27758.84	8.92	*a* ^3^P_2_—*w* ${\;}^{5}P_{3}^{{}^{\circ}}$	127	Pred	BK, U
3593.764	0*n*	27818.04	7.82	*a* ^3^H_4_—*v* ${\;}^{5}F_{5}^{{}^{\circ}}$	182	Pred	
3591.998	0	27831.72	1.73	*a* ^3^H_5_—*z* ${\;}^{1}G_{4}^{{}^{\circ}}$		New	
3589.586	2	27850.42	0.29	*b* ^3^G_4_—*u* ${\;}^{3}D_{3}^{{}^{\circ}}$		New	Z
3588.516	3	27858.72	8.69	*z* ${\;}^{7}P_{4}^{{}^{\circ}}$—*e* ^7^S_3_	394	Pred	BK, U
*3587.752*	3	27864.65	4.62	*a* ^3^D_1_—*t* ${\;}^{5}P_{1}^{{}^{\circ}}$		New	J
3586.740	3*n*	27872.52	2.51	*z* ${\;}^{7}F_{6}^{{}^{\circ}}$—*e* ^5^G_6_	325	SS	Z, ZZ
3582.324	2*n*	27906.87	6.77	*z* ${\;}^{5}D_{1}^{{}^{\circ}}$—*g* ^5^F_1_	568	Pred	BK, U
3579.829	1	27926.32	6.38	*z* ${\;}^{5}D_{3}^{{}^{\circ}}$—*e* ^3^G_4_	573	Pred	BK, Z
3575.754	1	27958.15	8.16	*b* ^3^G_3_—*u* ${\;}^{3}D_{2}^{{}^{\circ}}$		New	
3574.364	0	27969.02	9.03	*a* ^3^H_5_—*w* ${\;}^{5}G_{4}^{{}^{\circ}}$	181	Pred^b^	
3574.256	1	27969.86	9.94	*z* ${\;}^{5}D_{1}^{{}^{\circ}}$—*f* ^3^D_1_	574	New	
3567.748	1	28020.88	0.99	*z* ${\;}^{5}D_{3}^{{}^{\circ}}$—*f* ^5^G_3_	571	New	Z
3564.533	3	28046.16	6.34	*a* ^3^H_4_—*x* ${\;}^{3}G_{3}^{{}^{\circ}}$	183	Pred	BK, Z
3563.618	1	28053.36	3.48	*z* ${\;}^{7}F_{6}^{{}^{\circ}}$—*e* ^5^G_5_	325	Pred	U
3562.269	1*n*	28063.98	4.06	*a* ^3^D_3_— $54289_{3}^{{}^{\circ}}$		New	
3560.076	1	28081.27	1.37	*z* ${\;}^{7}F_{3}^{{}^{\circ}}$—*e* ^7^F_4_	321	Pred	
3551.114	1	28152.14	2.18	*z* ${\;}^{7}F_{4}^{{}^{\circ}}$—*e* ^7^F_3_	321	Pred	BK, Z
3530.976	1	28312.69	2.76	*a* ^3^P_0_—*v* ${\;}^{5}F_{1}^{{}^{\circ}}$	138	New	U
3528.942	1	28329.01	9.06	*a* ^5^F_5_—*z* ${\;}^{5}G_{4}^{{}^{\circ}}$	23	Pred^b^	
3528.316	0	28334.03	4.07	*a* ^3^F_3_—*z* ${\;}^{5}S_{2}^{{}^{\circ}}$?		New	
3528.233	1	28334.70	4.69	*a* ^3^H_4_—*v* ${\;}^{5}F_{3}^{{}^{\circ}}$	182	Pred	U
3515.404	1	28438.10	8.08	*b* ^3^F_2_—*z* ${\;}^{1}D_{2}^{{}^{\circ}}$	243	Pred	U
3509.736	3	28484.02	4.14	*z* ${\;}^{7}F_{0}^{{}^{\circ}}$—*f* ^5^F_1_	327	Pred	U
3507.139	3	28505.12	5.13	*z* ${\;}^{5}P_{2}^{{}^{\circ}}$—*i* ^5^D_2_	835	Pred	Z
3502.853	1	28539.99	0.01	*z* ${\;}^{5}D_{2}^{{}^{\circ}}$—*e* ^3^P_2_	577	Pred b	
3500.164	2	28561.92	2.03	*z* ${\;}^{7}F_{2}^{{}^{\circ}}$—*f* ^5^F_1_	327	New	U
3498.755	2	28573.42	3.47	*z* ${\;}^{7}F_{4}^{{}^{\circ}}$—*e* ^7^S_3_	330	New	W, Z
3487.138	0	28668.61	8.49	*c* ^3^F_3_— $62081_{2}^{{}^{\circ}}$?		New	
3481.292	1	28716.75	6.75	*c* ^3^P_0_—*v* ${\;}^{3}P_{1}^{{}^{\circ}}$	499	New	
*3473.303*	2	28782.80	2.84	*a* ^1^G_4_— $53358_{3}^{{}^{\circ}}$		New	V
3473.015	0*n*	28785.18	5.27	*z* ${\;}^{5}D_{2}^{{}^{\circ}}$—*f* ^3^F_3_	576	Pred	BK, U
3469.278	0	28816.19	6.22	*b* ^3^F_4_— $49457_{4}^{{}^{\circ}}$?		New	
3467.686	1	28829.42	9.43	*b* ^3^G_5_— *w* ${\;}^{3}H_{5}^{{}^{\circ}}$	442	New	
3456.374	1*n*	28923.77	3.76	*b ^3^*P_2_—*x* ${\;}^{1}D_{2}^{{}^{\circ}}$	375	New	
3448.190	1	28992.41	2.43	*a* ^3^H_6_—*z* ${\;}^{1}H_{5}^{{}^{\circ}}$	186	Pred	
3434.960	1*n*	29104.08	4.15	*a* ^1^D_2_—*t* ${\;}^{3}F_{2}^{{}^{\circ}}$	776	Pred	
3429.808	1	29147.79	$\left\{ \;\begin{array}{l} 7.75\\ 7.85 \end{array}\right.$	*a* ^1^G_4_—*y* ${\;}^{1}H_{5}^{{}^{\circ}}$	540	Pred	
				*b* ^3^F_2_—*w* ${\;}^{3}P_{2}^{{}^{\circ}}$	244	Pred	
3418.905	1	29240.74	0.62	*z* ${\;}^{7}P_{2}^{{}^{\circ}}$—*f* ^3^D_3_		New	W, Z
3414.564	1	29277.92	7.92	*b* ^3^H_4_—*s* ${\;}^{3}G_{4}^{{}^{\circ}}$?		New	U
3410.581	0*n*	29312.11	2.35	*b* ^3^F_3_—*w* ${\;}^{3}P_{2}^{{}^{\circ}}$	244	Pred	
3409.605	1	29320.50	0.66	*a* ^3^H_4_—*w* ${\;}^{3}F_{4}^{{}^{\circ}}$	188	New	U
3401.007	1	29394.62	4.71	*b* ^3^G_3_— $53734_{3}^{{}^{\circ}}$		New	U
3400.662	1	29397.60	7.71	*c* ^3^P_2_— $53734_{3}^{{}^{\circ}}$		New	BK, U
*3395.080*	1	29445.94	5.94	*b* ^3^G_3_ — $53785_{3}^{{}^{\circ}}$		New	V
3393.623	2	29458.58	8.60	*b* ^3^P_2_—*u* ${\;}^{3}D_{2}^{{}^{\circ}}$	376	(a, d)	} U, V
3393.590	1	29458.86	8.87	*a* ^3^G_3_—*y* ${\;}^{1}D_{2}^{{}^{\circ}}$	305	(a, d)	
3384.765	1	29535.67	5.39	*a* ^5^F_2_—*y* ${\;}^{3}F_{2}^{{}^{\circ}}$?	25	Pred b	
3381.990	1*n*	29559.90	9.89	*z* ${\;}^{7}P_{2}^{{}^{\circ}}$—*f* ^3^D_2_		New	
3381.498	1*n*	29564.20	4.19	*a* ^3^F_2_—*x* ${\;}^{5}P_{3}^{{}^{\circ}}$	49	New	
3375.724	1	29614.77	4.66	*b* ^3^G_4_— $53734_{3}^{{}^{\circ}}$		New	U
3374.176	2	29628.36	8.22	*a* ^5^P_1_—*y* ${\;}^{3}S_{1}^{{}^{\circ}}$	89	(a)	V
3370.254	0*n*	29662.83	2.77	*a* ^1^G_4_—*t* ${\;}^{3}G_{4}^{{}^{\circ}}$	542a	New	
3369.146	2	29672.59	2.64	*a* ^3^H_4_—*v* ${\;}^{3}G_{5}^{{}^{\circ}}$	191	Pred	Z
3364.402	1	29714.43	4.40	*a* ^1^G_4_— $54289_{3}^{{}^{\circ}}$		New	Z
*3358.911*	2	29763.00	3.06	*b* ^3^G_4_— $53882_{4}^{{}^{\circ}}$		New	V
3357.823	0	29772.64	2.69	*b* ^3^G_4_— $10_{3}^{{}^{\circ}}$	448	Pred^b^	U
*3356.695*	3	29782.65	2.71	*a* ^1^G_4_— $54357_{3}^{{}^{\circ}}$		New	V
3344.078	0	29895.01	4.93	*b* ^3^G_4_— $12_{5}^{{}^{\circ}}$	450	Pred	U
3337.915	1	29950.21	0.29	*b* ^3^G_3_— $54289_{3}^{{}^{\circ}}$		New	U
*3330.316*	1*n*	30018.55	8.60	*b* ^3^G_3_— $54357_{3}^{{}^{\circ}}$		New	V
3330.206	1	30019.54	9.48	*b* ^3^P_2_—*x* ${\;}^{3}S_{1}^{{}^{\circ}}$	378	New	U
*3329.970*	1*n*	30021.67	1.60	*c* ^3^P_2_— $54357_{3}^{{}^{\circ}}$		New	V
3316.558	1	30143.07	3.08	*a* ^5^P_3_—*w* ${\;}^{5}G_{3}^{{}^{\circ}}$?	86	New	U
3315.164	1	30155.74	5.69	*b* ^3^H_4_—*u* ${\;}^{3}F_{3}^{{}^{\circ}}$	618	Pred	
3313.555	0*n*	30170.38	0.24	*b* ^3^G_4_— $54289_{3}^{{}^{\circ}}$		New	
3304.346	1*n*	30254.46	4.37	*z* ${\;}^{5}F_{2}^{{}^{\circ}}$—*i* ^5^D_3_	710	Pred	U
3298.537	1	30307.74	7.80	*z* ${\;}^{5}F_{1}^{{}^{\circ}}$—*i* ^5^D_2_	710	New	U
3291.410	0	30373.37	3.10	*b* ^1^G_4_—*r* ${\;}^{3}G_{4}^{{}^{\circ}}$	954	Pred	U
3281.824	1	30462.08	2.04	*a* ^3^F_3_—*y* ${\;}^{5}G_{4}^{{}^{\circ}}$	50	Pred^b^	U
3276.978	0	30507.13	7.14	*z* ${\;}^{7}F_{5}^{{}^{\circ}}$—*e* ^5^H_6_	51	New	
3272.596	2	30547.98	7.99	*a* ^3^F_3_—*z* ${\;}^{5}H_{4}^{{}^{\circ}}$	95	Pred	Z
3269.416	2	30577.69	7.69	*a* ^5^P_2_—*x* ${\;}^{3}P_{2}^{{}^{\circ}}$		Pred	BK, U
3263.683	0	30631.40	1.31	*z* ${\;}^{7}F_{6}^{{}^{\circ}}$—*f* ^5^G_5_	680	New	U
3263.487	0	30633.24	3.62	*a* ^3^D_3_—*u* ${\;}^{3}F_{2}^{{}^{\circ}}$?	50	Pred	
3261.801	0	30649.07	9.09	*a* ^3^F_3_—*y* ${\;}^{5}G_{2}^{{}^{\circ}}$		New	BK, U
3258.627	1	30678.92	9.02	*z* ${\;}^{7}D_{1}^{{}^{\circ}}$—*f* ^5^D_2_	157	Pred	U
3249.504	1	30765.05	5.03	*a* ^3^F_3_—*z* ${\;}^{5}H_{3}^{{}^{\circ}}$	51	New	U
3241.502	0	30841.00	1.04	*a* ^5^F_1_—*y* ${\;}^{3}D_{1}^{{}^{\circ}}$	27	(a)	BK, U, W
3240.145	0*n*	30853.91	4.26	*z* ${\;}^{7}D_{3}^{{}^{\circ}}$—*e* ^7^P_3_	158	Pred	U
3238.313	0	30871.37	1.37	*a* ^1^G_4_—*v* ${\;}^{3}H_{4}^{{}^{\circ}}$	545	Pred	U
*3235.833*	1	30895.03	5.15	*b* ^3^P_2_— $53734_{3}^{{}^{\circ}}$		New	V
3235.312	1	30900.00	9.89	*a* ^3^G_4_—*y* ${\;}^{3}I_{5}^{{}^{\circ}}$	309	Pred	U
3232.155	1	30930.18	0.12	*b* ^3^F_2_—*u* ${\;}^{3}D_{3}^{{}^{\circ}}$	258	Pred^b^	U
3231.356	1	30937.83	7.71	*b* ^3^H_4_— $57565_{3}^{{}^{\circ}}$?		New	U
3230.085	1	30950.00	5.00	*a* ^5^F_3_—*y* ${\;}^{3}D_{2}^{{}^{\circ}}$	27	Pred	BK
*3229.595*	2*n*	30954.70	5.02	*z* ${\;}^{7}F_{5}^{{}^{\circ}}$—*g* ^7^D_5_?	333	New	V
3226.012	2	30989.08	9.09	*b* ^3^F_4_— $6_{5}^{{}^{\circ}}$		New	BK, Z
3223.480	1*n*	31013.42	3.42	*b* ^3^H_4_—*t* ${\;}^{3}F_{3}^{{}^{\circ}}$		New^c^	Z
3223.080	0	31017.27	7.33	*a* ^3^D_2_—*t* ${\;}^{3}F_{3}^{{}^{\circ}}$	682	Pred	U
3219.187	1*n*	31054.78	4.96	*a* ^3^P_2_—*w* ${\;}^{3}F_{2}^{{}^{\circ}}$?	141	New^c^	
3205.782	1	31184.63	4.66	*b* ^3^F_4_—*u* ${\;}^{3}G_{3}^{{}^{\circ}}$?	252	New	U
3204.306	1	31198.99	9.00	*b* ^3^H_5_—*t* ${\;}^{3}F_{4}^{{}^{\circ}}$		New	U
3199.920	1*n*	31241.75	1.65	*z* ${\;}^{7}D_{3}^{{}^{\circ}}$—*f* ^7^D_2_	156	New	U
3195.968	1	31280.38	0.49	*a* ^3^H_4_—*x* ${\;}^{3}H_{5}^{{}^{\circ}}$	192a	New	U
3193.726	0	31302.34	2.27	*a* ^3^D_1_—*t* ${\;}^{3}F_{2}^{{}^{\circ}}$	682	Pred	
*3188.026*	2	31358.31	8.36	*a* ^3^G_4_— $53358_{3}^{{}^{\circ}}$		New	Z
3187.171	1*n*	31366.72	6.87	*z* ${\;}^{7}F_{1}^{{}^{\circ}}$—*g* ^7^D_2_	333	Pred	BK, Z
3186.814	1	31370.23	0.25	*a* ^5^P_1_—*v* ${\;}^{3}D_{1}^{{}^{\circ}}$	100	Pred^b^	U
3184.112	1	31396.85	6.94	*z* ${\;}^{5}F_{5}^{{}^{\circ}}$—*g* ^5^G_5_	711	New	Z
3176.278	1*n*	31474.28	4.26	*z* ${\;}^{5}D_{2}^{{}^{\circ}}$—*i* ^5^D_3_	578	New	U
*3175.318*	1	31483.80	4.05	*a* ^3^G_3_— $53734_{3}^{{}^{\circ}}$?		New	U
3172.292	1	31513.83	3.82	*a* ^3^G_3_—*x* ${\;}^{1}F_{3}^{{}^{\circ}}$	312	Pred	U
3167.792	1	31558.60	8.73	*a* ^5^P_3_—*w* ${\;}^{3}F_{4}^{{}^{\circ}}$	99	Pred^b^	U
3166.982	1	31566.67	6.76	*b* ^3^G_3_—*s* ${\;}^{3}G_{4}^{{}^{\circ}}$	455	Pred^b^	
3166.259	2*n*	31573.88	4.05	*z* ${\;}^{7}D_{3}^{{}^{\circ}}$—*e* ^7^F_2_	155	Pred	Z
3161.558	1	31620.82	0.90	*a* ^3^H_4_— $4_{4}^{{}^{\circ}}$	195	Pred	Z
3159.437	1	31642.05	2.08	*a* ^3^G_3_— $10_{3}^{{}^{\circ}}$		New	U
3159.248	1	31643.94	3.91	*b* ^3^F_2_—*t* ${\;}^{3}D_{2}^{{}^{\circ}}$	259	Pred	U
3155.134	1*n*	31685.20	5.40	*z* ${\;}^{7}D_{1}^{{}^{\circ}}$—*f* ^5^F_2_	161	SS	Z, ZZ
3154.106	1	31695.53	5.56	*a* ^3^F_2_—*v* ${\;}^{5}D_{2}^{{}^{\circ}}$	53	Pred	U
3150.762	1	31729.17	9.20	*a* ^1^H_5_—*t* ${\;}^{3}H_{5}^{{}^{\circ}}$	813	New	U
3149.492	1	31741.96	1.93	*b* ^3^G_5_—*x* ${\;}^{1}H_{5}^{{}^{\circ}}$	453	Pred	U
3148.676	0	31750.18	0.08	*a* ^5^P_2_—*z* ${\;}^{1}D_{2}^{{}^{\circ}}$		New	U
3148.178	1*n*	31755.21	5.36	*a* ^3^G_3_— $11_{3}^{{}^{\circ}}$		New	
3144.924	1	31788.06	8.14	*a* ^3^H_5_— $4_{4}^{{}^{\circ}}$	195	Pred	U
3138.400	0	31854.14	4.18	*a* ^3^F_3_—*v* ${\;}^{5}D_{4}^{{}^{\circ}}$	53	Pred	
*3135.590*	1*n*	31882.69	2.74	*a* ^3^G_4_— $53882_{4}^{{}^{\circ}}$		New	Z
3134.641	0	31892.34	2.37	*a* ^3^G_4_— $10_{3}^{{}^{\circ}}$		New	U
3134.401	1	31894.78	4.67	*a* ^3^G_5_— $53610_{4}^{{}^{\circ}}$		New	Z
3133.174	0	31907.27	7.15	*a* ^5^P_3_— $49457_{4}^{{}^{\circ}}$?		New	
3131.238	0	31927.00	6.89	*a*^5^P_3_—*z* ${\;}^{1}D_{2}^{{}^{\circ}}$		New	U
3126.822	1	31972.09	1.94	*b* ^3^F_4_—*w* ${\;}^{3}H_{5}^{{}^{\circ}}$	260	Pred^b^	
3125.012	1	31990.60	0.49	*a* ^3^F_3_—*v* ${\;}^{5}D_{3}^{{}^{\circ}}$	53	Pred	U
3123.545	1*n*	32005.63	5.65	*a* ^3^G_4_— $11_{3}^{{}^{\circ}}$		New	Z
3121.151	1	32030.18	0.17	*z* ${\;}^{7}D_{1}^{{}^{\circ}}$—*g* ^5^D_2_	163	New	
3120.220	2*n*	32039.73	9.63	*a* ^3^G_3_— $54289_{3}^{{}^{\circ}}$		New	Z
3119.032	0	32051.94	1.90	*a* ^3^G_3_— $13_{4}^{{}^{\circ}}$	315a	Pred	U
3116.984	1*n*	32072.99	2.98	*z* ${\;}^{5}D_{3}^{{}^{\circ}}$—4_2_	578a	New	Z
3116.502	1*n*	32077.95	7.8	*a* ^5^P_2_—*x* ${\;}^{7}P_{3}^{{}^{\circ}}$?		New	BK, U
3116.379	1	32079.22	9.16	*b* ^3^F_3_—*s* ${\;}^{3}D_{3}^{{}^{\circ}}$	261	Pred b	U
3115.862	1	32084.54	4.53	*b* ^3^G_3_—*u* ${\;}^{3}H_{4}^{{}^{\circ}}$	456	New	U
3115.656	2	32086.66	6.59	*c* ^3^P_1_—*u* ${\;}^{3}F_{2}^{{}^{\circ}}$		New	Z
3114.054	1	32103.17	3.18	*a* ^3^F_3_—*v* ${\;}^{5}D_{2}^{{}^{\circ}}$	53	New	
*3113.592*	2	32107.93	7.94	*a* ^3^G_3_— $54357_{3}^{{}^{\circ}}$		New	Z
*3107.978*	2*n*	32165.93	6.13	*a* ^3^G_5_— $53882_{4}^{{}^{\circ}}$		New	Z
3103.760	1	32209.64	9.63	*a* ^3^P_1_—*x* ${\;}^{1}D_{2}^{{}^{\circ}}$		New	Z
3099.118	0	32257.88	7.92	*b* ^3^F_4_—*y* ${\;}^{3}I_{5}^{{}^{\circ}}$		New	
3098.963	1	32259.50	9.46	*a* ^5^P_1_—*w* ${\;}^{3}P_{2}^{{}^{\circ}}$	102	New	U, W
3097.500	0*n*	32274.73	4.82	*z* ${\;}^{7}D_{4}^{{}^{\circ}}$—*e* ^5^P_3_	165	Pred	Z
*3096.044*	1	32289.91	9.92	*a* ^3^G_4_— $54289_{3}^{{}^{\circ}}$		New	Z
3087.420	1*n*	32380.10	9.99	*z* ${\;}^{5}D_{3}^{{}^{\circ}}$—*g* ^5^G_4_?		New	U, W
3081.832	1	32438.81	8.87	*a* ^3^F_4_—*v* ${\;}^{5}D_{4}^{{}^{\circ}}$	53	Pred	BK, Z
3081.278	1	32444.64	4.53	*b* ^3^G_3_—*u* ${\;}^{3}F_{3}^{{}^{\circ}}$	457	New	BK, U
3071.276	1	32550.30	0.36	*b* ^3^G_5_—*u* ${\;}^{3}H_{6}^{{}^{\circ}}$	456	New^c^	BK, Z
*3056.250*	2	32710.33	0.37	*b* ^3^F_2_— $53749_{2}^{{}^{\circ}}$		New	BK
3030.605	2	32987.11	7.08	*a* ^3^P_2_—*v* ${\;}^{3}F_{3}^{{}^{\circ}}$	145	Pred^b^	BK, Z
3011.883	2	33192.15	2.08	*a* ^3^P_0_—*z* ${\;}^{1}P_{1}^{{}^{\circ}}$	UV135	New	BK, U, W
3006.598	0	33250.49	0.40	*b* ^3^F_4_— $10_{3}^{{}^{\circ}}$		New^c^	BK
2978.060	1	33569.11	9.25	*a* ^3^H_4_— $53358_{3}^{{}^{\circ}}$?		New	BK, W, Z
2975.655	0	33596.24	6.32	*b* ^3^F_4_—*t* ${\;}^{3}G_{4}^{{}^{\circ}}$		New	BK, U
2964.196	1	33726.11	5.83	*b* ^3^F_3_—*t* ${\;}^{3}G_{3}^{{}^{\circ}}$		New	BK, Z
2958.462	1	33791.48	1.55	*a* ^3^G_4_—*w* ${\;}^{1}F_{3}^{{}^{\circ}}$	317	New	BK, W, Z
2951.356	0	33872.83	2.85	*a* ^3^H_4_—*y* ${\;}^{1}F_{3}^{{}^{\circ}}$		New	BK, Z
2949.688	0*n*	33891.98	2.12	*a* ^5^P_2_—*u* ${\;}^{5}F_{3}^{{}^{\circ}}$	UV117	New	BK, Z
2947.116	0	33921.56	1.54	*b* ^3^H_4_—*t* ${\;}^{3}H_{5}^{{}^{\circ}}$	UV182	New	BK, Z
2946.095	1	33933.32	3.32	*a* ^3^F_2_—*y* ${\;}^{3}P_{1}^{{}^{\circ}}$		New	BK, Z
2945.870	0	33935.91	5.97	*b* ^3^H_4_— $60564_{3}^{{}^{\circ}}$		New c	BK, Z
*2945.050*	3	33945.36	5.23	*a* ^3^H_4_— $53734_{3}^{{}^{\circ}}$		New	G
2931.803	1	34098.73	8.68	*a* ^3^G_4_—*s* ${\;}^{3}G_{3}^{{}^{\circ}}$	UV166	(a)	BK, W, Z
2924.002	0	34189.70	9.79	*a* ^3^G_5_—*s* ${\;}^{3}G_{4}^{{}^{\circ}}$	UV166	New	BK, Z
2906.741	0	34392.72	2.74	*a* ^3^H_5_— $12_{5}^{{}^{\circ}}$	UV150	New	BK, U
2904.522	0	34418.99	8.93	*a* ^5^P_3_—*u* ${\;}^{3}D_{3}^{{}^{\circ}}$		New	BK
2898.867	1	34486.13	6.27	*a* ^5^P_2_—*t* ${\;}^{3}D_{3}^{{}^{\circ}}$		New	BK, W, Z
2897.635	1*n*	34500.79	0.81	*a* ^3^H_4_— $54289_{3}^{{}^{\circ}}$		(a, e)	BK, W, Z
2896.595	0	34513.18	3.08	*a* ^3^H_4_— $13_{4}^{{}^{\circ}}$		New	BK
*2891.904*	1*n*	34569.16	9.12	*a* ^3^H_4_— $54357_{3}^{{}^{\circ}}$		New	V
2891.705	1	34571.54	1.54	*b* ^3^F_3_—*v* ${\;}^{3}H_{4}^{{}^{\circ}}$	UV158	New^a^, ^e^	W, Z
2890.414	1	34586.98	7.06	*a* ^3^F_2_—*y* ${\;}^{3}S_{1}^{{}^{\circ}}$?		New	BK, Z
2882.634	0	34680.32	0.32	*a* ^3^H_5_— $13_{4}^{{}^{\circ}}$		New	
2879.741	0	34715.16	5.27	*b* ^3^F_2_—*w* ${\;}^{1}D_{2}^{{}^{\circ}}$		New^c^	BK, U
2878.962	1	34724.55	4.72	*a* ^3^F_2_—*w* ${\;}^{5}G_{3}^{{}^{\circ}}$	UV90	New	BK, U, W
*2878.762*	1	34726.97	6.99	*b* ^3^P_2_— $57565_{3}^{{}^{\circ}}$?		New	V
2876.725	1	34751.56	1.70	*b* ^3^F_2_—*w* ${\;}^{1}F_{3}^{{}^{\circ}}$?		New	BK, Z
2866.385	1	34876.91	6.99	*a* ^3^G_5_—*u* ${\;}^{3}F_{4}^{{}^{\circ}}$	UV168	New	BK, U
2861.996	0	34930.39	0.43	*a* ^5^P_1_—*x* ${\;}^{3}S_{1}^{{}^{\circ}}$		New	BK
2860.206	0*n*	34952.25	2.10	*a* ^3^G_4_—*v* ${\;}^{1}G_{4}^{{}^{\circ}}$		New	BK, Z
*2857.996*	1*n*	34979.28	9.31	*a* ^3^P_2_— $53358_{3}^{{}^{\circ}}$		New	Z
2830.754	0	35315.89	5.89	*a* ^3^G_3_— $57565_{3}^{{}^{\circ}}$?		New	BK, U, W
2819.462	1	35457.32	7.24	*a* ^3^D_2_— $62081_{2}^{{}^{\circ}}$?		(a, e)	BK, W, Z
2815.836	0	35502.98	2.92	*a* ^5^P_2_—*z* ${\;}^{1}P_{1}^{{}^{\circ}}$		New	BK, U
*2810.834*	1	35566.15	6.18	*a* ^3^G_4_— $57565_{3}^{{}^{\circ}}$		New	U, W
2802.285	0*n*	35674.65	4.78	*a* ^3^D_1_— $62081_{2}^{{}^{\circ}}$?		New	BK, Z
2797.046	0	35741.47	1.55	*b* ^3^F_4_—*u* ${\;}^{3}H_{5}^{{}^{\circ}}$		New^c^	BK
2790.762	0	35821.94	1.98	*a* ^5^P_1_— $53749_{2}^{{}^{\circ}}$		New^c^	BK
2783.560	0	35914.62	4.79	*a* ^3^F_3_—*w* ${\;}^{3}G_{3}^{{}^{\circ}}$	UV95	New	BK
2780.880	1	35949.23	9.20	*a* ^5^F_4_—*z* ${\;}^{5}H_{3}^{{}^{\circ}}$	UV45	New	BK, Z
2780.526	1	35953.81	3.78	*a* ^3^F_4_—*v* ${\;}^{5}F_{4}^{{}^{\circ}}$	UV92	New	BK, Z
2776.767	1	36002.48	2.44	*a* ^3^H_4_—*w* ${\;}^{1}F_{3}^{{}^{\circ}}$		New^c^	BK, U
*2772.511*	1	36057.74	7.72	*a* ^5^P_2_— $53785_{3}^{{}^{\circ}}$		New	V
2772.320	1	36060.23	0.23	*a* ^5^P_3_— $53610_{4}^{{}^{\circ}}$		New	BK, W, Z
2766.560	1	36135.30	5.38	*a* ^3^H_6_—*x* ${\;}^{1}H_{5}^{{}^{\circ}}$	UV152	New	BK
2760.623	1	36213.01	3.07	*a* ^5^P_3_—*x* ${\;}^{1}F_{3}^{{}^{\circ}}$	UV127	New	BK
2759.500	0	36227.74	7.81	*c* ^3^P_2_— $60564_{3}^{{}^{\circ}}$?		New	BK, U
2758.993	1	36234.40	4.53	*a* ^5^P_3_— $53785_{3}^{{}^{\circ}}$		New	BK, W
2751.808	1	36329.00	9.09	*a* ^3^F_2_—*v* ${\;}^{3}D_{1}^{{}^{\circ}}$?		New	BK, W, Z
2749.688	0	36357.01	7.14	*a* ^5^F_1_—*y* ${\;}^{5}S_{2}^{{}^{\circ}}$	UV49	New	BK, Z
2745.952	0	36406.48	6.37	*a* ^3^F_4_—*z* ${\;}^{1}H_{5}^{{}^{\circ}}$		New	BK, Z
2732.778	1	36581.97	2.05	*b* ^3^G_5_—*t* ${\;}^{3}H_{6}^{{}^{\circ}}$?		New	BK
2725.311	1	36682.20	2.00	*a* ^3^F_3_—*w* ${\;}^{3}F_{3}^{{}^{\circ}}$	UV98	New	BK, W, Z
2724.344	1*n*	36695.22	5.00	*z* ${\;}^{7}D_{3}^{{}^{\circ}}$—2_4_	UV144	New	BK, U
2723.032	0	36712.90	2.97	*a* ^3^H_5_—*u* ${\;}^{3}H_{6}^{{}^{\circ}}$	UV154	New	BK
*2707.451*	2	36924.16	4.21	*b* ^3^F_4_— $57565_{3}^{{}^{\circ}}$		New	Z
2679.513	0	37309.13	9.21	*c* ^3^P_1_— $62081_{2}^{{}^{\circ}}$?		New	BK, U
*2667.22*	1	37481.08	1.10	*a* ^3^F_4_— $49457_{4}^{{}^{\circ}}$?		New^c^	U
2648.548	1	37745.30	5.47	*c* ^3^P_2_— $62081_{2}^{{}^{\circ}}$?		New	BK, W, Z
2648.164	1	37750.77	0.80	*a* ^3^F_4_—*y* ${\;}^{3}H_{4}^{{}^{\circ}}$	UV99	New^c^	BK, W, Z
2647.390	1	37761.81	1.81	*a* ^3^H_4_—*t* ${\;}^{3}F_{4}^{{}^{\circ}}$		New	BK, U
2642.274	0	37834.92	4.96	*a* ^5^F_3_—*y* ${\;}^{3}G_{3}^{{}^{\circ}}$	UV51	New	BK, U
2641.031	1	37852.72	2.78	*a* ^3^H_4_—*t* ${\;}^{3}F_{3}^{{}^{\circ}}$		New	BK, U
2627.230	1	38051.55	1.68	*a* ^5^F_4_—*y* ${\;}^{3}G_{4}^{{}^{\circ}}$	UV51	New	BK, Z
2627.118	1	38053.18	3.06	*a* ^3^F_3_—*x* ${\;}^{1}G_{4}^{{}^{\circ}}$		New	BK, W, Z
*2609.220*	2	38314.19	4.15	*a* ^3^G_3_— $60564_{3}^{{}^{\circ}}$		New	G
2603.042	0	38405.12	5.11	*a* ^3^P_2_—*u* ${\;}^{3}F_{3}^{{}^{\circ}}$		New	BK
2596.618	0	38500.12	0.18	*a* ^5^F_5_—*y* ${\;}^{3}G_{4}^{{}^{\circ}}$	UV51	New	BK, U
2596.077	1	38508.15	8.22	*a* ^3^G_3_—*t* ${\;}^{3}H_{4}^{{}^{\circ}}$	UV171	New	BK, U
2593.268	0	38549.86	0.01	*a* ^3^G_4_—*t* ${\;}^{3}H_{5}^{{}^{\circ}}$	UV171	New	BK, U
*2592.285*	3	38564.47	4.44	*a* ^3^G_4_— $60564_{3}^{{}^{\circ}}$		New	G
2588.898	0	38614.92	5.09	*a* ^5^F_2_—*z* ${\;}^{3}S_{1}^{{}^{\circ}}$		New	BK, U
2580.939	1	38733.99	3.86	*a* ^5^F_1_—*u* ${\;}^{5}D_{2}^{{}^{\circ}}$	UV55	New	BK, Z
2580.561	1	38739.67	9.76	*a* ^3^F_2_—*y* ${\;}^{1}D_{2}^{{}^{\circ}}$		New^c^	BK, U
2580.281	0	38743.87	3.70	*a* ^3^F_3_—*v* ${\;}^{3}F_{4}^{{}^{\circ}}$?		New	BK, Z
*2554.518*	1	39134.59	4.41	*b* ^3^P_1_— $62081_{2}^{{}^{\circ}}$?		New	G
2550.812	1	39191.44	1.45	*a* ^5^F_2_—*u* ${\;}^{5}D_{1}^{{}^{\circ}}$	UV55	New	BK, Z
2547.468	0	39242.88	2.91	*b* ^3^P_2_— $62081_{2}^{{}^{\circ}}$?		New	BK, U
2546.176	1	39262.80	2.84	*a* ^3^P_2_—*t* ${\;}^{3}F_{3}^{{}^{\circ}}$		New	BK, U
2544.462	1	39289.24	9.17	*a* ^5^F_3_—*w* ${\;}^{3}D_{3}^{{}^{\circ}}$	UV58	New	BK, U
2538.693	1	39378.52	8.47	*a* ^5^F_3_—*z* ${\;}^{3}H_{4}^{{}^{\circ}}$	UV57	New^e^	BK, Z
*2529.306*	2	39524.66	4.59	(*b* ^3^F_2_ — $60564_{3}^{{}^{\circ}}$)?		New^f^	Z
*2518.824*	2	39689.12	9.09	*b* ^3^F_3_— $60564_{3}^{{}^{\circ}}$		New	Z
2512.266	3	39792.72	2.57	*a* ^5^F_5_—*u* ${\;}^{5}D_{4}^{{}^{\circ}}$	UV55	New	BK, U
2509.390	1	39838.32	8.33	*a* ^5^P_2_— $57565_{3}^{{}^{\circ}}$		New	BK, U
2508.948	1	39845.34	5.40	*a* ^5^F_2_—*w* ${\;}^{5}G_{2}^{{}^{\circ}}$	UV59	New	BK, Z
2504.635	1	39913.95	4.04	*a* ^5^P_2_—*t* ${\;}^{3}F_{3}^{{}^{\circ}}$		New	BK, U
2504.101	1	39922.46	2.47	*b* ^3^F_4_— $60564_{3}^{{}^{\circ}}$		New	BK, Z
2499.693	1	39992.86	2.88	*a* ^3^F_4_—*u* ${\;}^{3}D_{3}^{{}^{\circ}}$	UV104	New	BK, U
2498.698	1	40008.78	8.77	*a* ^5^F_1_—*v* ${\;}^{5}P_{2}^{{}^{\circ}}$		New	BK, U
2494.504	1	40076.04	6.00	*a* ^5^F_4_—*z* ${\;}^{1}G_{4}^{{}^{\circ}}$	UV61	New	BK, U
2492.822	1	40103.08	3.13	*a* ^5^F_3_—*w* ${\;}^{5}G_{2}^{{}^{\circ}}$	UV59	New	BK, U
2489.917	1	40149.87	9.98	*a* ^5^F_1_—*x* ${\;}^{3}P_{2}^{{}^{\circ}}$	UV65	New	BK
2484.530	1	40236.92	7.03	*a* ^3^F_4_—*t* ${\;}^{3}D_{3}^{{}^{\circ}}$		New^c^	BK, Z
2480.393	1	40304.02	4.09	*a* ^5^F_2_—*v* ${\;}^{5}P_{1}^{{}^{\circ}}$		New	BK, U
2469.666	1	40479.07	9.09	*a* ^5^D_3_—*z* ${\;}^{5}S_{2}^{{}^{\circ}}$	UV10	New	BK, U
2466.530	1	40530.53	0.35	*a* ^5^F_2_—*x* ${\;}^{3}P_{1}^{{}^{\circ}}$	UV65	New	BK, U
2460.069	1	40636.97	6.82	*a* ^3^F_4_—*w* ${\;}^{3}H_{5}^{{}^{\circ}}$		New	BK, U
2456.704	1	40692.63	2.56	*a* ^3^F_2_—*y* ${\;}^{1}F_{3}^{{}^{\circ}}$	UV106	New	BK
2453.568	3	40744.64	4.66	*a* ^3^H_5_—*t* ${\;}^{3}H_{6}^{{}^{\circ}}$	UV157	New	BK, U
2452.345	1	40764.95	4.94	*a* ^3^F_2_— $53734_{3}^{{}^{\circ}}$		New	BK, U
2452.172	2	40767.83	7.92	*a* ^3^F_3_— $9_{4}^{{}^{\circ}}$	UV105	New	BK, U
*2451.384*	2	40780.93	0.82	*a* ^3^F_2_— $53749_{2}^{{}^{\circ}}$		New^c^	G
*2450.439*	3	40796.66	6.58	*a* ^3^F_3_— $53358_{3}^{{}^{\circ}}$		New	G
2448.570	0	40827.80	7.73	*a* ^3^F_3_—*t* ${\;}^{5}P_{3}^{{}^{\circ}}$?		New	BK
2444.905	1	40889.00	8.95	*a* ^3^H_4_—*q* ${\;}^{3}G_{5}^{{}^{\circ}}$?		New	BK, U
2439.630	3	40977.40	7.42	*a* ^3^F_4_—*s* ${\;}^{3}D_{3}^{{}^{\circ}}$		New	BK, U
2439.169	1	40985.14	5.14	*a* ^5^F_4_—*w* ${\;}^{3}G_{4}^{{}^{\circ}}$	UV64	New	BK, U
2433.056	2	41088.11	7.96	*a* ^5^F_1_—*v* ${\;}^{3}D_{2}^{{}^{\circ}}$	UV68	New^c^	BK, U
2432.402	0	41099.16	8.96	*a* ^5^F_4_—*w* ${\;}^{3}G_{3}^{{}^{\circ}}$	UV64	New	BK
2432.332	1	41100.34	0.18	*a* ^3^F_3_—*y* ${\;}^{1}F_{3}^{{}^{\circ}}$	UV106	New	BK
2430.192	2	41136.53	6.64	*a* ^3^H_5_—*t* ${\;}^{3}H_{4}^{{}^{\circ}}$	UV157	New^c^	BK, Z
2429.431	1	41149.41	9.28	*a* ^5^F_2_—*v* ${\;}^{3}D_{3}^{{}^{\circ}}$	UV68	New	BK, W
2426.313	1*n*	41202.29	2.33	*a* ^3^F_3_—*x* ${\;}^{1}F_{3}^{{}^{\circ}}$		New	BK, Z
2414.318	0	41406.98	7.01	*a* ^5^F_3_—*v* ${\;}^{3}D_{3}^{{}^{\circ}}$	UV68	New	BK
2412.766	1	41433.61	3.64	*a* ^5^F_5_—*w* ${\;}^{3}G_{4}^{{}^{\circ}}$	UV64	New	BK
2412.172	0	41443.81	3.87	*a* ^3^F_3_— $11_{3}^{{}^{\circ}}$		New	BK, U
2411.968	1	41447.32	7.38	*a* ^5^F_2_—*w* ${\;}^{3}F_{2}^{{}^{\circ}}$	UV67	New	BK, U
2411.558	1	41454.36	4.35	*a* ^5^F_5_—*z* ${\;}^{1}H_{5}^{{}^{\circ}}$		New	BK
2401.136	1	41634.28	4.18	*a* ^3^F_4_— $53610_{4}^{{}^{\circ}}$		New	BK
2394.058	0	41757.36	7.25	*a* ^3^F_4_— $53734_{3}^{{}^{\circ}}$		New	BK
2393.094	1	41774.18	4.29	*a* ^5^F_5_—*y* ${\;}^{1}G_{4}^{{}^{\circ}}$	UV66	New	BK
2391.826	1	41796.33	6.45	*a* ^3^F_3_— $54357_{3}^{{}^{\circ}}$		New	BK, Z
2387.830	1	41866.27	6.18	*a* ^5^F_4_—*w* ${\;}^{3}F_{3}^{{}^{\circ}}$	UV67	New	BK, U
2385.580	1	41905.75	5.65	*a* ^3^F_4_— $53882_{4}^{{}^{\circ}}$		New	BK, Z
2378.604	1	42028.64	8.56	*a* ^3^F_4_— $11_{3}^{{}^{\circ}}$		New	BK
2376.971	0	42057.51	7.45	*a* ^5^F_2_—*w* ${\;}^{3}P_{1}^{{}^{\circ}}$?		New	BK
2375.678	0	42080.40	0.58	*a* ^5^F_4_— $49457_{4}^{{}^{\circ}}$?		New	BK, U
2367.394	0	42227.64	7.67	*a* ^5^F_4_—*y* ${\;}^{3}H_{5}^{{}^{\circ}}$		New	BK
2362.624	1	42312.89	2.83	*a* ^3^F_4_— $54289_{3}^{{}^{\circ}}$		New	BK
2361.936	1	42325.21	5.10	*a* ^3^F_4_— $13_{4}^{{}^{\circ}}$		New	BK
2356.196	1	42428.31	8.1	*a* ^5^F_4_—*x* ${\;}^{7}P_{3}^{{}^{\circ}}$?		New^c^	BK
2351.884	1	42506.10	$\{\;\begin{array}{c} 5.92\\ 6.04 \end{array}$	*a* ^5^F_5_—*y* ${\;}^{3}H_{6}^{{}^{\circ}}$	UV12	} New	BK
				*a* ^5^D_2_—*y* ${\;}^{5}G_{2}^{{}^{\circ}}$			
2350.626	1	42528.84	8.78	*a* ^3^P_1_— $62081_{2}^{{}^{\circ}}$?		New	BK
2346.304	1	42607.18	7.06	*a* ^5^D_3_—*y* ${\;}^{5}G_{4}^{{}^{\circ}}$	UV12	New^c^	BK
2345.018	1*n*	42630.54	0.2	*a* ^5^F_5_—*x* ${\;}^{7}P_{4}^{{}^{\circ}}$?		New	BK, U, W
2331.088	1	42885.27	5.11	*a* ^3^F_3_—*v* ${\;}^{3}H_{4}^{{}^{\circ}}$	UV108	New^c^	BK
2323.627	1	43022.96	3.00	*a* ^5^D_4_—*y* ${\;}^{5}G_{4}^{{}^{\circ}}$	UV12	New	BK, U
2296.188	2	43537.03	6.90	*a* ^3^F_3_—*s* ${\;}^{3}G_{3}^{{}^{\circ}}$	UV111	New	BK, X, Z
2295.310	1	43553.68	3.61	*a* ^5^F_1_—*y* ${\;}^{1}D_{2}^{{}^{\circ}}$		New	BK, U
2294.100	3	43576.65	6.58	*a* ^5^F_3_—*v* ${\;}^{3}F_{4}^{{}^{\circ}}$		New	BK, U
2290.907	3	43637.38	7.23	*a* ^5^F_3_—*v* ${\;}^{3}F_{3}^{{}^{\circ}}$		New	BK
2288.608	1	43681.21	1.11	*a* ^5^F_3_— $4_{4}^{{}^{\circ}}$	UV72	New	BK
2287.462	3	43703.09	3.05	*a* ^3^P_2_— $62081_{2}^{{}^{\circ}}$?		New	BK, U
2286.442	3	43722.59	2.53	*a* ^5^F_2_—*y* ${\;}^{1}D_{2}^{{}^{\circ}}$		New	BK, Z
2281.629	2	43814.81	4.76	*a* ^3^F_2_—*u* ${\;}^{3}F_{3}^{{}^{\circ}}$	UV112	New^a^,^e^	U, X
2279.152	2	43862.42	2.38	*a* ^3^F_3_—*u* ${\;}^{3}H_{4}^{{}^{\circ}}$		New	BK, U
2275.758	3	43927.83	7.87	*a* ^5^F_4_—*v* ${\;}^{3}F_{4}^{{}^{\circ}}$		New	BK, U
2275.676	1	43929.42	9.30	*a* ^3^F_4_—*s* ${\;}^{3}G_{4}^{{}^{\circ}}$	UV111	New	
2273.893	1	43963.86	3.91	*a* ^5^F_3_—*u* ${\;}^{5}P_{3}^{{}^{\circ}}$	UV73	New	BK, U
2272.610	3	43988.68	8.52	*a* ^5^F_4_—*v* ${\;}^{3}F_{3}^{{}^{\circ}}$		New	BK, X, Z
2270.675	3	44026.16	6.10	*a* ^5^F_1_—*t* ${\;}^{3}D_{1}^{{}^{\circ}}$		New	BK, U
2270.368	1	44032.11	2.40	*a* ^5^F_4_— $4_{4}^{{}^{\circ}}$?	UV72	New^c^	BK, Z
2256.063	3	44311.28	1.16	*a* ^5^F_2_—*u* ${\;}^{3}D_{2}^{{}^{\circ}}$	UV75	New	BK
2231.090	2	44807.22	7.07	*a* ^3^F_4_—*u* ${\;}^{3}F_{3}^{{}^{\circ}}$	UV112	New	U
2222.059	2	44989.30	9.14	*a* ^3^F_3_—*t* ${\;}^{3}F_{4}^{{}^{\circ}}$	UV114	New	BK, U
2208.714	1	45261.10	0.96	*a* ^5^D_2_—*x* ${\;}^{5}G_{2}^{{}^{\circ}}$	UV20	New	BK, U
2192.819	3	45589.15	9.09	*a* ^3^F_4_— $57565_{3}^{{}^{\circ}}$?		New	BK, X, Z
2190.879	3	45629.52	9.46	*a* ^5^F_3_— $53358_{3}^{{}^{\circ}}$		New	BK, U
2189.720	1	45653.66	3.65	*a* ^5^F_1_—*v* ${\;}^{3}P_{1}^{{}^{\circ}}$?		New	BK, U
2185.216	0	45747.75	7.71	*a* ^5^F_2_— $53734_{3}^{{}^{\circ}}$		New	BK, X, Z
*2184.46*	1	45763.58	3.59	*a* ^5^F_2_— $53749_{2}^{{}^{\circ}}$		New	Z
2178.797	2	45882.52	2.37	*a* ^5^F_3_— $53610_{4}^{{}^{\circ}}$		New	BK, U
2177.690	1	45905.84	5.74	*a* ^5^F_2_— $10_{3}^{{}^{\circ}}$	UV80	New	BK, U
2174.142	3	45980.74	0.75	*a* ^5^F_4_— $53358_{3}^{{}^{\circ}}$		New	BK, X, Z
2172.332	0	46019.05	9.02	*a* ^5^F_2_— $11_{3}^{{}^{\circ}}$	UV82	New	BK
2172.221	1	46021.40	1.32	*a* ^5^F_3_— $53749_{2}^{{}^{\circ}}$		New	BK
2170.554	3	46056.74	6.67	*a* ^5^F_3_— $53785_{3}^{{}^{\circ}}$		New	BK, X, Z
2167.271	1	46126.50	6.50	*a* ^5^F_2_—*t* ${\;}^{5}P_{2}^{{}^{\circ}}$	UV78	New	BK
2165.982	3	46153.95	3.84	*a* ^5^F_3_— $53882_{4}^{{}^{\circ}}$		New	BK
2162.243	3	46233.75	3.66	*a* ^5^F_4_— $53610_{4}^{{}^{\circ}}$		New	BK, X, Z
2158.993	1	46303.34	3.29	*a* ^5^F_2_— $54289_{3}^{{}^{\circ}}$		New	BK
2158.732	3	46308.94	8.94	*a* ^5^D_1_—*x* ${\;}^{3}F_{2}^{{}^{\circ}}$	UV25	New	BK
2156.504	3	46356.78	6.73	*a* ^5^F_4_— $53734_{3}^{{}^{\circ}}$?		New^d^	BK, X, Z
2155.816	3	46371.57	1.50	*a* ^5^F_2_— $54357_{3}^{{}^{\circ}}$		New	BK, X, Z
2155.114	0	46386.67	6.50	*a* ^5^F_4_—*x* ${\;}^{1}F_{3}^{{}^{\circ}}$		New	BK
*2154.127*	3	46407.92	7.96	*a* ^5^F_4_— $53785_{3}^{{}^{\circ}}$		New	U
2149.620	1	46505.22	5.13	*a* ^5^F_4_— $53882_{4}^{{}^{\circ}}$		New	BK, X, Z
2147.039	3	46561.12	1.02	*a* ^5^F_3_— $54289_{3}^{{}^{\circ}}$		New	BK, X, Z
2143.892	3	46629.45	9.33	*a* ^5^F_3_— $54357_{3}^{{}^{\circ}}$		New	BK, Z
*2141.471*	3	46682.16	2.16	*a* ^5^F_5_— $53610_{4}^{{}^{\circ}}$		New	Z
*2130.962*	3	46912.35	2.31	*a* ^5^F_4_— $54289_{3}^{{}^{\circ}}$		New	
2127.863	2	46980.67	0.62	*a* ^5^F_4_— $54357_{3}^{{}^{\circ}}$		New	BK, X, Z
2127.467	1	46989.41	9.29	*a* ^5^D_2_—*w* ${\;}^{5}G_{3}^{{}^{\circ}}$	UV28	New	BK, U
2124.494	0	47055.16	5.02	*a* ^5^F_5_—*t* ${\;}^{3}G_{5}^{{}^{\circ}}$	UV81	New	BK, U
2123.118	0	47085.65	5.50	*a* ^5^F_5_— $12_{5}^{{}^{\circ}}$		New	BK, U
2111.220 (vac.)	0	47350.98	0.77	*a* ^5^D_1_—*v* ${\;}^{5}F_{2}^{{}^{\circ}}$	UV31	New	BK, N, U
*1899.21*	1	52653.4	3.53	*a* ^5^D_2_— $53358_{3}^{{}^{\circ}}$		New	N
*1891.74*	10	52861.5	1.26	*a* ^5^D_1_— $53749_{2}^{{}^{\circ}}$		New	N
*1883.91*	2	53081.2	0.74	*a* ^5^D_2_— $53785_{3}^{{}^{\circ}}$		New	N
*1865.30*	15	53610.6	0.44	*a* ^5^D_4_— $53610_{4}^{{}^{\circ}}$		New	N
*1859.26*	2	53784.9	4.79	*a* ^5^D_4_— $53785_{3}^{{}^{\circ}}$		New	N

a Improved wavelength of previously known line listed here as preferable to the value quoted in Table B of the Monograph.[1]

b Predicted line not listed in Table C of the Monograph. See Ref. 3.

c Blend of Fe I and Fe II. See J. C. Dobbie, Ann. Solar Phys. Obs. Cambridge, **V**, pt. 1, 1938.

d Member of resolved doublet.

e Designation in Table B of the Monograph to be rejected.

f This line is listed as 10*r* III by A. S. King; the line classified here may be masked.

**Table 4:** New *Fe I* lines, unclassified

Wavelength A	Intensity	Wave Number cm^−1^	Notes and References
			
8558.647	1	11680.88	
7952.338	1	12571.46	
7945.090	2	12582.93	
7937.914	1*n*	12594.30	
7854.150	0*n*	12728.62	
7775.383	1	12857.56	
7714.603	0	12958.86	
7702.968	0	12978.44	
7693.734	0	12994.01	
7675.972	1	13024.08	
7674.966	0	13025.79	
7657.254	0	13055.92	
7643.394	0	13079.59	
7626.473	0	13108.61	
7624.011	0	13112.85	
7615.529	0	13127.45	
7614.148	0	13129.83	
7600.948	0	13152.63	
7599.624	0	13154.92	
7596.842	0	13159.74	
7588.834	0	13173.63	
7580.647	0	13187.86	
7576.505	0	13195.06	
7573.444	0	13200.40	U
7567.043	0	13211.56	
7554.743	0	13233.07	
7528.735	0	13278.79	
7523.388	0	13288.22	
7501.061	0	13327.78	
7476.098	0	13372.28	
7453.230	1	13413.31	
7438.336	1	13440.16	
7435.798	0*n*	13444.75	
7214.630	1	13856.91	
7192.458	1*n*	13899.62	
7009.530	1	14262.36	
6864.229	1	14564.26	
6692.280	1	14938.47	
6587.388	1	15176.33	
6515.104	1*n*	15344.71	
6491.395	1	15400.75	
6478.828	1	15430.63	
6452.148	1*n*	15494.43	
6392.988	3	15637.82	
6366.040	3	15704.01	
6347.162	3	15750.72	
6333.354	3	15785.06	
6242.738	3	16014.18	
6143.052	3	16274.05	
5884.323	1	16989.60	
5699.308	2	17541.12	
5644.033	3	17712.91	
5625.704	3	17770.62	
5601.298	2	17848.05	
5590.661	3	17882.01	
5589.852	2*n*	17884.60	
5582.673	2*n*	17907.59	
5553.176	2*n*	18002.71	
5532.856	2	18068.83	
5521.096	2	18107.32	U, W
5512.658	3*n*	18135.03	
5477.744	1*n*	18250.6	
5458.572	2	18314.72	
5418.598	2*n*	18449.83	W
5351.751	1	18680.28	
5350.798	1	18683.60	
5350.434	0	18684.88	
5333.396	2	18744.57	
5319.079	2*n*	18795.02	
5317.854	1	18799.35	
5313.542	1*n*	18814.60	
5312.539	0	18818.16	
5312.024	0	18819.98	
5311.641	1	18821.34	
5310.855	0*n*	18824.12	
5310.270	0*n*	18826.20	
5309.780	0	18827.93	U, W
5308.228	0	18833.44	
5307.736	1	18835.18	
5306.060	0*n*	18841.13	
5303.874	1*n*	18848.90	
5301.408	1	18857.67	
5199.090	3	19228.78	
5182.983	2	19288.54	
5181.335	3	19294.67	T, U
5176.806	1	19311.55	
5173.498	3	19323.90	
5166.694	3	19349.35	
5160.094	3*n*	19374.10	
5154.039	2*n*	19396.86	
5149.746	3	19413.02	
5147.107	2	19422.98	
5140.826	3	19446.71	
5097.498	3	19612.00	
5025.514	3	19892.91	
5013.914	3	19938.94	U
4996.792	3	20007.26	
4996.174	0*n*	20009.73	
4992.502	1	20024.45	
4974.246	0	20097.94	U, W
4961.040	3	20151.44	U, W
4948.299	1	20203.33	
4947.645	3*n*	20206.00	U
4947.418	0	20206.92	
4944.306	3	20219.64	
4932.134	3	20269.54	
4924.333	3	20301.65	
4915.806	3	20336.87	
4913.168	3	20347.78	
4911.587	3	20354.34	U
4906.144	0	20376.92	
4902.368	1	20392.61	
4901.272	2	20397.17	
4900.816	2	20399.07	
4900.520	0	20400.30	
4898.930	0	20406.92	
4895.672	0	20420.50	
4880.976	2	20482.82	
4875.081	3	20506.75	
4864.512	2	20551.31	
4862.992	3	20557.73	
4861.947	3	20562.15	U, W
4833.817	1	20681.81	
4832.036	1	20689.43	
4825.357	3	20718.07	U, W
4821.572	2	20734.33	
4818.038	1	20749.54	
4814.365	2	20765.37	
4811.407	0	20778.13	
4805.072	2*n*	20805.53	
4793.336	0	20856.47	
4784.034	2	20897.02	
4782.147	1*n*	20905.26	
4776.451	3*n*	20930.19	
4774.939	1*n*	20936.82	
4768.697	3	20964.23	U
4766.821	3	20972.48	
4756.888	0	21016.27	
4756.356	1	21018.62	
4752.470	1	21035.81	
4746.278	0	21063.25	
4733.945	0	21118.12	
4730.009	3*n*	21135.70	
4728.160	1	21143.96	
4722.522	0*n*	21169.20	
4719.716	0	21181.79	
4719.238	0*n*	21183.94	
4717.620	2*n*	21191.20	
4717.358	2*n*	21192.38	
4716.483	0	21196.31	
4712.515	1	21214.16	
4710.650	0	21222.56	
4703.228	0	21256.04	
4702.642	0	21258.69	
4702.299	0*n*	21260.24	
4701.849	0*n*	21262.28	BK
4699.424	3*n*	21273.25	
4691.769	0	21307.96	
4688.964	0	21320.71	
4686.641	0	21331.27	
4686.348	0*n*	21332.61	
4685.583	0*n*	21336.09	
4684.662	2	21340.28	
4680.628	1	21358.68	
4676.757	0	21376.36	
4674.297	2	21387.60	U, W
4671.337	1*n*	21401.16	
4664.750	1	21431.38	
4660.920	1*n*	21448.99	
4660.478	1*n*	21451.02	
4656.548	1*n*	21469.12	
4650.388	0*n*	21497.56	
4648.986	0	21504.05	(c)
4640.958	1	21541.24	U, W
4640.340	2*n*	21544.11	U, W
4605.610	2*n*	21706.57	BK, U
4597.403	2	21745.32	U, W
4590.815	2*n*	21776.52	U
4585.337	1	21802.54	
4582.297	1*n*	21817.00	
4581.186	1	21822.29	
4566.940	3	21890.36	U, W
4560.892	1	21919.39	
4557.287	2	21936.73	U
4533.078	2*n*	22053.88	U
4517.136	1	22131.71	
4509.804	2*n*	22167.70	U, W
4509.430	2	22169.53	
4500.652	1*n*	22212.77	
4480.731	1*n*	22311.53	(c)
4478.649	1*n*	22321.90	
4471.600	0	22357.09	
4468.452	0	22372.84	
4465.552	0	22387.36	
4460.642	0	22412.01	
4452.773	1*n*	22451.61	
4451.684	0*n*	22457.11	
4445.050	0	22490.62	W
4432.087	1*n*	22556.40	
4431.799	1*n*	22557.87	U
4425.090	0*n*	22592.07	
4424.608	1	22594.53	
4424.061	1	22597.32	
4420.266	2*n*	22616.72	
4416.688	0	22635.04	W
4416.423	1	22636.40	U
4416.137	0	22637.87	
4412.146	1	22658.34	
4411.914	2*n*	22659.54	
4406.946	1	22685.08	
4399.260	0	22724.71	
4395.937	0*n*	22741.89	U
4394.098	1	22751.41	
4385.776	1*n*	22794.58	
4384.848	2	22799.40	
4381.429	1*n*	22817.19	
4380.108	1	22824.07	
4378.486	2	22832.53	
4376.446	1	22843.17	
4374.988	1	22850.78	
4374.116	1	22855.34	
4372.558	1	22863.48	
4371.742	0	22867.75	
4364.772	1	22904.27	
4362.484	1	22916.28	
4357.940	0	22940.17	
4357.098	0	22944.61	
4355.144	1	22954.90	
4347.000	0	22997.91	
4344.928	1	23008.87	U
4341.392	1	23027.61	
4340.950	0	23029.96	
4340.279	0	23033.52	
4337.455	0	23048.51	
4337.366	1	23048.99	
4336.122	1	23055.60	
4335.773	0	23057.46	U, W
4335.399	0	23059.44	
4334.928	1*n*	23061.95	
4334.428	0*n*	23064.61	
4332.711	1	23073.75	U
4332.432	1	23075.24	U
4331.411	1*n*	23080.68	U
4330.640	1	23084.78	
4327.410	0	23102.01	
4323.658	1*n*	23122.06	
4320.767	1*n*	23137.53	U
4319.231	0	23145.76	
4316.832	0	23158.62	
4316.525	0	23160.27	
4315.495	0	23165.80	
4315.400	0	23166.31	
4313.812	1	23174.84	W
4312.950	1*n*	23179.47	
4310.531	1	23192.48	U
4309.704	0	23196.92	
4306.991	1	23211.54	BK
4306.068	0	23216.51	
4302.541	0	23235.54	
4302.344	0	23236.61	
4299.770	1	23250.52	
4298.324	1	23258.34	
4297.106	1*n*	23264.93	
4293.612	1*n*	23283.86	U
4293.400	0*n*	23285.01	U
4288.297	1	23312.72	
4287.952	1	23314.60	
4285.984	1	23325.30	
4283.770	1	23337.36	BK
4282.914	1*n*	23342.02	
4282.052	1*n*	23346.72	
4281.921	1*n*	23347.43	
4281.855	1	23347.79	
4281.490	1	23349.78	BK
4280.285	1*n*	23356.36	U
4278.604	1*n*	23365.53	
4276.082	1	23379.32	
4272.528	1	23398.76	U
4270.838	1	23408.02	
4270.628	1*n*	23409.17	
4270.251	1*n*	23411.24	
4269.730	1*n*	23414.10	U
4252.911	1*n*	23506.69	
4251.999	1*n*	23511.73	
4251.657	0*n*	23513.62	U
4251.288	1*n*	23515.66	
4249.596	1*n*	23525.03	U
4249.216	1	23527.13	
4236.461	1	23597.96	
4234.122	1*n*	23611.00	
4229.406	1	23637.32	U
4228.384	1*n*	23643.04	U
4223.221	1	23671.94	
4219.850	1	23690.85	W
4219.018	1	23695.52	
4218.880	1	23696.30	
4218.812	1	23696.68	
4218.464	1	23698.64	
4211.568	1	23737.44	
4210.868	1	23741.38	
4209.447	1	23749.40	
4206.210	1*n*	23767.68	U
4203.258	1*n*	23784.37	U
4199.557	1*n*	23805.33	
4197.512	0	23816.93	
4194.099	1	23836.31	
4193.600	1	23839.14	U, W
4192.794	0	23843.72	
4192.622	1*n*	23844.70	
4192.374	1*n*	23846.11	
4191.974	1*n*	23848.39	
4190.938	1*n*	23854.28	
4190.000	1*n*	23859.62	
4189.015	1*n*	23865.24	
4188.300	1*n*	23869.31	
4185.793	3	23883.60	
4184.546	1	23890.72	
4184.400	1	23891.56	
4181.278	1*n*	23909.39	U
4180.176	0	23915.70	
4179.689	1	23918.48	
4174.208	0*n*	23949.89	
4160.333	1*n*	24029.76	U
4158.366	1	24041.13	U
4157.306	1	24047.26	
4156.322	1*n*	24052.95	U
4155.914	1	24055.31	U
4152.778	1	24073.48	
4152.651	1	24074.21	
4148.794	1*n*	24096.60	
4141.400	1*n*	24139.62	U
4139.718	1	24149.42	U
4139.276	1	24152.00	
4137.642	1	24161.54	U, W
4136.200	1*n*	24169.96	U
4135.887	1	24171.79	
4135.607	1*n*	24173.43	U
4135.039	1*n*	24176.75	
4131.146	1*n*	24199.53	
4127.252	1	24222.36	
4127.120	1	24223.14	
4126.965	1	24224.05	
4124.332	0*n*	24239.51	
4119.746	1*n*	24266.50	
4118.065	1*n*	24276.40	U
4110.814	0	24319.22	
4110.310	1	24322.20	U
4107.160	1	24340.86	
4107.015	1*n*	24341.72	
4096.695	1	24403.03	
4095.346	1	24411.07	U
4094.422	1	24416.58	U
4086.406	1	24464.48	U
4081.264	1	24495.30	BK, U, W
4072.332	0	24549.02	
4072.183	0	24549.92	
4071.282	0	24555.35	U
4071.146	0	24556.17	
4070.954	1	24557.33	
4070.554	0	24559.74	
4070.090	0	24562.54	
4069.786	0	24564.38	
4069.700	0	24564.90	
4069.610	1	24565.44	
4068.898	0	24569.74	
4068.800	0	24570.33	
4068.704	0	24570.91	
4068.483	0	24572.25	
4068.254	0	24573.63	
4066.768	1	24582.61	
4061.848	1*n*	24612.38	
4054.454	1	24657.27	BK, U
4049.924	1	24684.85	U
4041.828	1*n*	24734.29	
4037.136	1*n*	24763.04	U
4036.552	0*n*	24766.62	
4033.648	1	24784.45	U
4026.770	1	24826.78	
4022.564	0	24852.74	
4021.374	1	24860.10	
4021.002	1	24862.40	
4015.023	1	24899.42	
4014.340	1	24903.66	
4014.072	1	24905.32	
4012.628	1*n*	24914.28	U
4010.618	1	24926.77	BK, U
4010.522	1	24927.36	
4009.388	1	24934.41	
4009.240	1*n*	24935.33	
4008.531	1	24939.74	
4003.287	1*n*	24972.41	
3999.398	1	24996.70	
3996.540	1	25014.57	
3996.139	1	25017.08	
3995.456	0*n*	25021.36	
3993.642	1	25032.72	U
3991.395	1*n*	25046.81	
3983.645	0	25095.54	
3983.518	1*n*	25096.34	U
3978.247	1	25129.59	
3977.420	1	25134.82	U
3970.863	1	25176.32	
3966.973	1*n*	25201.01	
3962.717	1*n*	25228.07	Z
3960.642	1	25241.29	
3957.836	1	25259.18	
3946.007	1	25334.90	BK, U, W
3943.166	1*n*	25353.16	U
3940.422	1	25370.81	U
3939.730	1	25375.27	
3936.558	1	25395.71	U
3934.976	1	25405.92	
3934.356	1*n*	25409.93	U, W
3932.266	1*n*	25423.43	Z
3931.883	0	25425.91	U
3926.422	1*n*	25461.27	U
3924.032	1*n*	25476.78	
3915.256	1	25533.88	BK, U?, W
3904.564	1*n*	25603.80	
3843.528	0*n*	26010.38	
3842.796	0	26015.34	
3842.556	0	26016.96	
3838.567	1	26044.00	
3838.201	0	26046.48	
3838.084	1	26047.28	U^c^
3837.914	1	26048.43	U
3819.679	1	26172.78	U, W
3819.354	0	26175.01	
3819.276	2	26175.54	
3818.950	3	26177.78	
3818.815	0*n*	26178.70	
3818.593	2*n*	26180.23	Z
3818.141	1	26183.32	
3818.043	0	26184.00	U
3817.793	2	26185.71	
3817.424	1	26188.24	
3817.268	0	26189.31	
3817.094	3*n*	26190.51	
3816.702	1	26193.20	
3816.594	1	26193.94	
3815.188	1*n*	26203.59	U
3815.021	1	26204.74	
3814.892	1	26205.62	
3814.668	2	26207.16	
3814.268	1	26209.91	
3814.044	2	26211.45	
3812.761	2	26220.27	
3812.624	1	26221.21	
3812.518	2	26221.94	U
3812.306	1*n*	26223.40	
3812.026	2	26225.33	
3811.484	3	26229.06	U
3811.101	1	26231.69	W
3810.526	2	26235.65	U
3810.332	0*n*	26236.98	U
3809.882	0	26240.08	
3809.334	0	26243.86	
3808.871	0	26247.05	
3807.986	1	26253.15	
3807.791	0*n*	26254.49	U
3807.313	0	26257.79	
3807.088	0	26259.34	
3806.476	0	26263.56	U
3806.052	0	26266.49	W
3805.771	3	26268.43	U
3805.568	0	26269.83	U
3805.478	1	26270.45	
3805.140	1	26272.78	
3805.024	1	26273.58	
3804.842	0*n*	26274.84	
3804.501	1	26277.20	
3804.228	0	26279.08	
3803.704	0	26282.70	U
3803.408	3	26284.75	
3803.106	1*n*	26286.84	
3802.936	0*n*	26288.01	
3802.682	1	26289.76	
3802.536	0	26290.78	
3801.468	1	26298.16	
3801.066	0	26300.94	U
3800.955	0	26301.71	
3800.813	0*n*	26302.69	
3800.699	2	26303.48	U
3800.407	1	26305.50	
3800.269	1	26306.46	
3800.173	0	26307.12	
3800.026	0	26308.14	
3799.208	1	26313.80	
3799.109	1	26314.49	
3799.024	0	26315.08	
3798.910	1	26315.87	
3798.196	0	26320.82	
3798.072	1	26321.68	U
3797.666	3	26324.49	
3797.191	0	26327.78	
3797.054	0	26328.73	
3796.730	0	26330.98	
3796.608	1*n*	26331.82	U, W
3796.259	0	26334.24	
3795.824	0	26337.26	U
3795.741	1	26337.84	U
3795.608	0	26338.76	
3795.386	1	26340.30	
3794.562	1	26346.02	
3794.486	1	26346.55	
3794.174	0	26348.72	
3793.726	3	26351.83	
3793.136	1	26355.93	
3792.454	0	26360.67	U, W
3792.292	0	26361.79	U
3791.930	0	26364.31	
3790.917	0*n*	26371.35	U
3790.254	3	26375.97	
3788.944	1*n*	26385.08	U
3788.593	0	26387.53	
3788.472	0	26388.37	
3788.281	1	26389.70	
3787.661	1	26394.02	
3787.379	0	26395.99	U
3787.059	0	26398.22	
3786.907	0	26399.28	U
3786.811	2	26399.95	
3786.443	3	26402.51	
3785.486	3	26409.19	
3785.262	0	26410.75	U
3785.127	0	26411.69	
3784.934	0	26413.04	
3784.812	1	26413.89	
3784.698	1	26414.68	
3784.386	3*n*	26416.86	U
3783.721	2	26421.51	
3783.470	0	26423.26	
3783.275	1	26424.62	
3782.862	3	26427.50	U
3781.749	0	26435.28	
3781.300	0	26438.42	
3780.966	0	26440.76	
3780.828	0	26441.72	
3780.678	0	26442.77	
3780.562	0	26443.58	
3780.256	0	26445.72	U
3780.004	0	26447.49	
3779.810	0	26448.84	U
3778.928	0	26455.02	
3778.809	1	26455.85	U
3777.848	1	26462.58	
3777.588	0*n*	26464.40	
3777.186	3	26467.22	U
3776.838	0	26469.66	
3776.226	0	26473.94	
3776.110	0	26474.76	
3775.972	0	26475.73	
3775.656	2	26477.94	
3775.503	0	26479.02	
3775.364	0	26479.99	
3775.080	0	26481.98	U
3774.952	1	26482.88	
3774.180	0	26489.57	
3773.999	0	26493.28	
3773.470	0	26494.16	
3773.166	1	26496.75	
3772.841	0	26497.70	
3772.702	0	26498.67	
3772.287	0	26501.59	U
3771.816	1*n*	26504.90	
3771.249	0*n*	26508.88	
3770.786	0	26512.14	
3770.548	0	26513.81	
3769.310	1*n*	26522.52	
3769.071	1*n*	26524.20	
3768.697	0	26526.83	U
3768.561	3	26527.79	
3767.788	1	26533.23	
3767.610	3	26534.49	
3764.718	0	26554.87	
3759.732	1	26590.08	
3754.068	0	26630.20	
3741.903	0*n*	26716.77	
3741.743	0*n*	26717.92	Z?
3728.972	2	26809.42	U
3723.946	1	26845.60	
3723.570	0*n*	26848.31	
3706.070	1	26975.08	U
3699.810	1	27020.72	U
3695.632	2*n*	27051.27	Z
3694.752	1	27057.72	
3680.962	2*n*	27159.08	U
3680.396	2n	27163.26	Z
3676.008	0	27195.68	
3672.114	0	27224.52	U
3668.730	1*n*	27249.63	U, W
3665.845	1	27271.07	U, W
3665.762	1	27271.69	
3659.214	1	27320.49	
3659.094	1*n*	27321.39	
3658.742	1	27324.02	U
3653.157	0*n*	27365.79	U
3652.969	0	27367.20	
3651.918	1*n*	27375.07	U
3651.182	1*n*	27380.59	
3646.580	1*n*	27415.14	U
3642.198	0*n*	27448.13	
3640.834	1	27458.41	
3640.096	1	27463.98	U
3639.964	1*n*	27464.97	U
3639.605	1*n*	27467.68	
3639.502	1*n*	27468.46	U
3639.308	1*n*	27469.92	U
3628.868	1	27548.95	W
3628.620	1	27550.83	
3628.414	0	27552.40	S, U
3628.210	0	27553.94	
3627.419	1*n*	27559.95	
3625.498	1	27574.56	
3622.981	1	27593.71	
3622.751	1*n*	27595.46	
3622.431	3	27597.90	
3620.679	1	27611.26	
3619.942	2*n*	27616.88	U
3617.629	1*n*	27634.53	
3617.472	1	27635.73	
3617.007	2	27639.29	BK, U
3616.857	0	27640.43	
3616.722	1	27641.46	
3616.642	2	27642.08	
3616.036	0	27646.71	U, W
3615.814	2	27648.40	U
3615.518	0	27650.67	
3615.328	0	27652.12	Z
3614.404	0*n*	27659.91	
3614.272	1	27660.20	BK
3613.296	0	27667.67	
3612.783	1	27671.60	
3612.671	0	27672.46	
3612.418	0	27674.40	
3612.233	2	27675.81	
3611.935	1*n*	27678.10	
3611.768	1	27679.38	
3611.651	1*n*	27680.27	
3611.519	1	27681.28	
3611.392	0*n*	27682.26	
3611.188	1*n*	27683.82	U
3610.972	0	27685.48	
3610.867	0	27686.28	
3609.996	1	27692.96	
3609.884	1*n*	27693.82	
3609.426	1	27697.34	U
3608.365	1	27705.48	
3607.780	2*n*	27709.97	
3607.634	1*n*	27711.09	
3607.333	1	27713.41	U
3607.256	1	27714.00	
3607.102	2*n*	27715.18	
3606.932	1	27716.49	
3606.253	2	27721.70	U
3606.165	1	27722.38	
3606.016	0	27723.53	
3604.874	0	27732.31	
3604.090	0*n*	27738.34	
3603.956	2	27739.37	Z
3603.449	1	27743.28	
3603.350	2	27744.04	
3602.898	0	27747.52	
3602.323	0	27751.95	
3602.223	0	27752.72	
3601.858	1	27755.53	
3601.273	1	27760.04	
3601.116	1	27761.25	
3600.870	1	27763.15	
3600.675	1	27764.65	U
3600.533	1	27765.74	U
3600.418	1	27766.63	U
3600.036	1*n*	27769.58	U
3599.972	2*n*	27770.07	Z
3599.842	1	27771.07	
3599.760	1	27771.71	
3599.468	0	27773.96	U
3599.293	0	27775.31	
3598.554	0	27781.01	
3598.233	1*n*	27783.49	
3597.804	1	27786.80	BK, W
3597.478	0	27789.32	
3597.314	0	27790.59	
3597.158	0	27791.79	
3596.853	1*n*	27794.15	
3596.727	0	27795.12	
3596.612	0	27796.01	
3596.438	0	27797.36	
3596.334	0	27798.16	
3595.526	1	27804.41	U
3595.438	0	27805.09	
3594.427	1	27812.91	
3594.312	0	27813.80	
3594.176	1	27814.85	U
3593.860	0	27817.30	
3593.119	2	27823.03	
3592.354	1	27828.96	
3591.940	0	27832.17	
3591.174	0	27838.10	
3590.500	1	27843.33	
3590.422	0	27843.93	
3590.204	0	27845.62	
3589.876	0*n*	27848.17	
3589.224	2	27853.23	
3588.724	0	27857.11	
3588.284	2	27860.52	
3587.844	0	27863.94	
3587.604	0	27865.80	
3587.527	1	27866.40	
3587.328	3	27867.95	
3586.390	0*n*	27875.24	
3586.332	0	27875.69	U
3584.468	0	27890.18	
3584.354	1	27891.07	
3584.264	1*n*	27891.77	
3584.110	1	27892.97	
3583.921	1*n*	27894.44	
3583.687	2	27896.26	Z
3583.577	1	27897.12	
3583.036	0	27901.33	
3582.970	3*n*	27901.84	
3582.908	1	27902.32	
3582.460	2	27905.81	
3582.032	1*n*	27909.15	
3581.951	1	27909.78	
3580.402	1	27921.85	U
3579.562	1	27928.41	Z
3577.490	1*n*	27944.58	
3574.609	2	27967.10	
3555.736	1	28115.54	
3550.309	0	28158.52	
3550.189	0	28159.47	
3539.376	1	28245.50	
3538.688	1	28250.99	BK
3525.622	1	28355.68	U
3520.668	0	28395.58	
3520.214	0	28399.24	
3519.500	1	28405.00	
3519.350	0	28406.22	U
3515.534	1	28437.05	
3515.286	1	28439.06	BK, U, W
3510.682	2	28476.35	U
3506.946	1	28506.68	
3506.092	1	28513.63	
3501.375	1	28552.04	U
3499.271	1	28569.21	
3499.154	1	28570.16	
3492.310	1*n*	28626.15	
3491.780	0	28630.50	U
3488.827	2	28654.73	
3486.142	1	28676.80	
3485.766	1	28679.89	(c)
3484.586	2	28689.60	
3483.890	2	28695.33	U
3482.446	1	28707.23	(c)
3472.318	0*n*	28790.96	U, W
3457.894	1	28911.05	
3455.726	0	28929.19	
3455.393	1*n*	28931.98	
3450.743	1	28970.96	
3448.606	1*n*	28988.92	
3447.700	1	28996.54	
3444.532	2	29023.20	
3435.219	0*n*	29101.88	
3434.182	0	29110.67	
3430.554	1*n*	29141.46	
3430.066	0*n*	29145.60	
3429.179	1	29153.14	
3425.441	1	29184.95	
3423.558	1	29201.00	
3422.120	2	29213.27	Z
3421.930	1	29214.90	
3420.864	1	29224.00	
3420.250	0*n*	29229.25	
3419.258	1*n*	29237.73	
3416.840	1	29258.42	U
3416.562	1	29260.80	
3414.432	0	29279.05	
3412.418	1	29296.33	
3412.134	1	29298.77	
3409.742	1	29319.32	U
3409.461	0	29321.74	
3408.474	1	29330.23	U, W
3402.743	1*n*	29379.62	
3398.945	1*n*	29412.45	U
3398.620	1	29415.26	
3398.374	1	29417.40	(c)
3395.692	0	29440.63	U
3395.436	0	29442.85	
3393.053	1*n*	29463.53	
3390.218	0	29488.16	
3387.558	1	29511.32	
3384.946	0*n*	29534.09	
3384.392	0	29538.92	
3381.132	1	29567.40	
3380.756	1	29570.69	U
3379.688	1	29580.04	
3379.409	1*n*	29582.48	U
3377.971	3	29595.07	V
3377.833	0	29596.28	
3377.345	0*n*	29600.56	
3377.006	0*n*	29603.53	
3375.372	2	29617.86	V
3375.046	1	29620.72	U
3373.750	0	29632.10	
3373.300	1	29636.05	U
3371.928	1	29648.11	
3371.526	2	29651.64	
3371.304	1	29653.60	U
3371.200	1	29654.51	
3369.958	0	29665.44	
3368.800	1*n*	29675.64	
3368.556	0	29677.78	
3368.435	0	29678.85	(c)
3367.660	1	29685.68	U
3367.292	1	29688.92	U
3366.494	0	29695.96	
3364.492	0	29713.63	
3359.608	1	29756.83	U
3359.390	1	29758.76	U
3358.573	1	29766.00	
3358.386	2*n*	29767.65	
3358.320	1	29768.24	
3358.044	0	29770.68	
3357.558	1	29774.99	Z
3354.854	1*n*	29798.99	
3354.512	1	29802.03	
3354.176	1	29805.02	
3352.004	1	29824.33	U
3351.854	1	29825.66	
3345.234	0	29884.68	
3341.626	0	29916.95	
3341.507	0	29918.01	
3340.292	0	29928.90	
3340.184	0	29929.86	U
3332.840	0*n*	29995.81	
3328.589	0	30034.12	
3328.470	1*n*	30035.19	U
3324.142	0	30074.30	
3323.454	0	30080.52	
3323.352	0*n*	30081.45	
3316.838	0	30140.52	U
3311.307	0	30190.87	
3311.200	0*n*	30191.84	U, W
3310.916	0*n*	30194.43	U
3309.660	1*n*	30205.89	U
3309.283	1*n*	30209.33	U
3305.572	1*n*	30243.24	
3305.376	0*n*	30245.04	
3305.258	0*n*	30246.12	
3303.918	0	30258.38	
3303.046	1*n*	30266.37	
3301.350	0*n*	30281.92	
3297.728	0*n*	30315.18	
3297.414	0	30318.06	
3295.897	0	30332.02	
3295.316	0	30337.37	U
3294.963	0*n*	30340.62	W
3294.829	0	30341.85	
3294.621	0*n*	30343.77	U
3294.267	0	30347.03	U
3287.483	1*n*	30409.65	U^c^
3284.202	0	30440.03	
3282.440	1	30456.37	Z
3270.620	1	30566.43	
3270.312	0*n*	30569.31	
3268.885	1	30582.66	
3263.989	0	30628.53	
3263.805	0	30630.25	
3263.062	1	30637.23	Z
3262.878	1	30638.96	U
3262.132	0	30645.96	
3261.636	0	30650.62	U
3261.425	0	20652.60	
3261.146	0	30655.23	
3260.880	0	30657.73	U
3260.723	0	30659.20	
3260.624	0	30660.14	
3260.549	0	30660.84	
3260.460	0	30661.68	S
3259.708	1	30668.75	U
3258.092	1	30683.96	U
3255.091	0	30712.25	(c)
3250.212	0*n*	30758.35	U
3249.844	1	30761.83	W, Z
3241.378	1*n*	30842.18	U
3232.656	1	30925.39	
3232.347	0	30928.34	U
3222.808	0*n*	31019.88	U
3220.486	1*n*	31042.25	U
3216.581	0*n*	31079.93	
3216.343	0	31082.23	
3204.454	0*n*	31197.55	U
3203.677	0*n*	31205.12	
3202.958	2	31212.12	Z
3202.188	0*n*	31219.62	U
3200.908	0	31232.11	
3200.092	1	31240.07	U
3198.492	1	31255.70	Z
3195.235	1*n*	31287.56	W, Z
3192.521	2	31314.16	Z
3189.612	0	31342.71	U
3186.276	0	31375.53	U
3184.215	1	31395.84	U
3181.142	0*n*	31426.16	
3170.978	0	31526.89	
3165.280	0	31583.64	U
3163.494	0	31601.47	
3163.372	0	31602.69	U
3158.193	0	31654.51	Z
3157.293	1	31663.54	U
3146.915	0	31767.95	U
3146.270	1	31774.46	U
3145.431	1	31782.94	
3139.485	1	31843.13	U
3137.238	0	31865.94	U
3133.732	1	31901.59	U^c^
3133.440	0	31904.56	U
3132.660	1	31912.50	Z
3130.972	0	31929.71	U
3129.800	0	31941.66	U
3125.390	0	31986.73	
3119.864	0*n*	32043.39	
3115.954	0	32083.60	
3109.614	1*n*	32149.01	Z
3107.322	1*n*	32172.72	Z
3053.781	0*n*	32736.77	BK
3049.564	1*n*	32782.04	BK, U
3049.356	1*n*	32784.27	BK, W, Z
3038.334	0*n*	32903.20	BK, U
3012.942	1*n*	33180.48	BK
3010.198	1*n*	33210.73	BK^c^
2995.256	0	33376.40	BK, W, Z
2985.750	0	33482.65	BK, Z
2979.867	0	33548.75	BK, W, Z
2975.298	0	33600.27	BK, U
2971.776	0	33640.09	BK, W, Z
2963.518	0*n*	33733.83	BK, Z
2962.585	1*n*	33744.45	BK, W, Z
2956.377	0	33815.30	BK, U
2955.619	0	33823.98	BK, U
2954.957	0	33831.55	BK, U
2945.702	1	33937.84	BK, W, Z
2934.598	0	34066.25	BK, U
2933.051	1	34084.22	BK
2930.395	1	34115.11	BK, U
2915.658	0	34287.54	BK, U
2896.772	0	34511.07	BK
2880.398	0	34707.24	BK, U
2877.724	0	34739.49	BK
2877.582	0	34741.21	BK
2862.080	0	34929.37	
2841.260	1	35185.31	BK, U, W
2830.090	0*n*	35324.17	BK^c^
2829.485	0*n*	35331.72	BK, W
2829.312	0*n*	35333.89	BK, U
2808.665	1	35593.62	BK, W, Z
2801.922	0	35679.27	BK, U
2785.002	1*n*	35896.03	BK
2783.845	0	35910.94	BK
2775.818	1*n*	36014.79	BK, U, W
2765.254	1	36152.36	BK, U, W^c^
2764.128	1*n*	36167.09	BK, U^c^
2762.939	0	36182.65	BK, U
2758.093	0	36246.22	BK, U
2757.558	0	36253.26	BK, U
2751.325	1*n*	36335.38	BK, Z?
2731.883	0	36593.96	BK^c^
2730.265	0	36615.64	BK
2723.892	1*n*	36701.30	(c)
2715.503	0	36814.68	BK^c^
2713.510	0*n*	36841.72	BK, U^c^
2704.798	0	36960.38	BK, U
2702.622	0*n*	36990.13	BK
2685.140	1	37230.95	BK, U, W^c^
2684.536	0	37239.33	BK, U
2676.162	1*n*	37355.84	BK
2674.625	1	37377.31	BK, U
2673.552	0	37392.31	BK
2672.831	0*n*	37402.40	BK
2668.904	1*n*	37457.43	BK, U, W^c^
2668.724	1	37459.95	BK
2665.814	0	37500.84	BK, U, W
2664.184	1*n*	37523.78	BK^c^
2663.169	0	37538.08	BK, U
2658.699	1	37601.19	BK, U
2658.111	0	37609.51	BK
2656.337	0	37634.63	BK, U
2652.212	0	37693.16	BK, U
2648.914	0	37740.08	BK
2642.403	0	37833.07	(c)
2618.850	1	38173.31	BK
2610.464	0	38295.93	BK, U
2609.600	1*n*	38308.61	BK
2597.443	0	38487.90	BK, U
2591.782	0	38571.96	BK, U
2581.754	0*n*	38721.77	BK, U
2569.322	1*n*	38909.12	BK, Z^c^
2568.584	1	38920.29	BK, U
2563.990	1	38990.02	BK, U^c^
2561.068	2	39034.51	
2559.080	0	39064.83	BK, U
2558.118	1	39079.52	BK, U
2557.982	1	39081.60	BK, U
2557.792	1*n*	39084.50	BK, U
2557.020	0*n*	39096.30	BK
2554.218	0*n*	39139.18	BK, U
2532.269	1	39478.41	BK, U, W
2531.449	1*n*	39491.20	BK, W, Z^a^,^e^
2527.262	1	39556.62	BK
2524.603	1	39598.28	BK
2513.246	1	39777.20	
2502.503	3	39947.95	BK, U
2494.797	1*n*	40071.34	BK, U
2492.236	1*n*	40112.51	BK
2485.435	0	40222.26	BK, W
2485.206	1*n*	40225.97	BK
2480.732	0*n*	40298.51	BK
2478.300	1*n*	40338.06	BK
2477.304	1*n*	40354.27	BK^c^
2476.464	1*n*	40367.96	BK, U
2475.758	1	40379.47	BK
2475.466	2	40384.23	BK
2475.018	1*n*	40391.54	BK, U
2467.953	1*n*	40507.16	BK, U
2467.573	2*n*	40513.40	BK, U
2466.348	1*n*	40533.52	BK, U
2466.093	0	40537.71	BK, U
2464.349	1	40566.40	BK, U
2462.963	1	40589.23	BK, U
2456.147	2*n*	40701.86	BK, U
2454.399	1	40730.84	BK, U
2452.965	1	40754.65	BK
2451.697	3n	40775.73	BK, W
2448.826	1*n*	40823.53	BK, U
2440.335	2	40965.56	BK, U
2436.072	2	41037.24	BK, U
2419.236	1	41322.81	BK, U
2413.764	1	41416.48	BK, U
2411.738	1*n*	41451.27	BK
2405.302	0	41562.18	BK, U
2403.542	0*n*	41592.61	BK, U
2402.938	1	41603.06	BK, U
2402.109	1	41617.42	(c)
2398.726	1	41676.11	BK
2396.102	0*n*	41721.74	BK, U^c^
2395.186	1	41737.70	BK, U
2394.303	0*n*	41753.09	BK
2388.090	1	41861.71	BK
2385.386	1	41909.16	BK, U
2383.790	1*n*	41937.22	BK, U
2376.276	0	42069.81	BK
2375.990	0	42074.88	BK
2360.755	2	42346.38	BK, U
2358.622	2	42384.68	BK
2341.373	1	42696.90	BK, U
2335.246	1	42808.91	BK, U
2333.024	1	42849.68	BK
2329.093	0	42922.00	BK
2326.047	0	42978.20	BK, U
2324.970	0	42998.11	BK, U
2324.260	0	43011.24	BK
2323.416	1	43026.86	BK, U
2323.286	0	43029.27	BK, U
2322.684	0	43040.42	BK^c^
2321.583	0	43060.83	BK, U
2321.220	0	43067.57	BK, U^c^
2321.029	0	43071.11	BK
2319.680	1*n*	43096.16	BK, U
2319.441	0	43100.60	BK
2316.290	0	43159.22	BK
2314.337	0	43195.64	BK
2313.650	1	43208.47	BK, U
2310.732	0	43263.02	BK, U^c^
2304.544	1	43378.99	BK, U
2300.242	3	43460.30	BK
2274.288	1	43956.22	BK
2223.747	1	44955.16	BK
2213.828	3	45156.56	U
2208.280	0	45270.00	BK, U
2206.444	1	45307.66	BK, U
2200.494	3	45430.16	BK, U
2200.084	1	45438.62	U
2189.514	1	45657.96	BK, U
2186.760	2	45715.45	BK
2176.670	3	45927.35	BK, U
2176.216	1	45936.93	BK, U
2175.825	3	45945.18	BK, X
2173.451	1	45995.36	BK
2172.658	3*n*	46012.15	BK
2172.037	1	46025.30	BK
2156.432	3	46358.32	X, Z^d^
2151.005	2	46475.28	BK
2146.806	3	46566.17	BK, U
2146.458	1	46573.72	BK
2143.369	2	46640.83	BK, U
2142.820	1	46652.78	BK, U
2142.575	1	46658.11	BK, U
2135.300	0*n*	46817.06	BK
2132.526	0*n*	46877.95	BK, U
2124.948	2*n*	47045.11	BK, U
2121.864	1*n*	47113.48	BK, U

a Improved wavelength of previously known line listed here as preferable to the value quoted in Table B of the Monograph.[1]

b Predicted line not listed in Table C of the Monograph. See Ref. 3.

c Blend of Fe I and Fe II. See J. C. Dobbie, Ann. Solar Phys. Obs. Cambridge, **V**, pt. 1, 1938.

d Member of resolved doublet.

e Designation in Table B of the Monograph to be rejected.

f This line is listed as 10*r* III by A. S. King; the line classified here may be masked.

**Table 5:** Faint lines of *Fe I* in the solar spectrum

Laboratory		Sun			
Wavelength A	Intensity	Wavelength A	Intensity Δλ/λ	⊙—Lab. A	Solar Identification
					
8126.520	1	8126.48	0.6	0.04	Fe I?
8090.341	1	8090.464	3.7	+0.123	Atm Fe I?
7714.603	0	7714.59	1.2	−0.01	Fe I?
7588.834	0	7588.849	1.2	+0.015	Fe I?
7438.336	1	7438.38	0.2	+0.04	Fe I?
7214.630	1	7214.60	0.1	−0.03	Atm? Fe I?
7192.458	1*n*	7192.465	4.4	+0.007	Fe I –| Atm
7092.866	1*n*	7092.848	1.1	−0.018	Fe I
6692.280	1	6692.304	0.3	+0.024	Fe I?
5899.094	2	5899.106	0.1	+0.012	Fe I
5699.308	2	5699.322	1.2	+0.014	Fe I
5644.033	3	5644.037	2.5	+0.004	Fe I
5625.704	3	5625.687	*5.2*	−0.017	–Fe I
5589.852	2*n*	5589.861	2.9	+0.009	Fe I
5458.572	2	5458.58	1.1	+0.01	Fe I
5350.798	1	5350.789	0.7	−0.009	Fe I
5350.434	0	5350.454	0.2	+0.020	Fe I?
5308.228	0	5308.212	0.1	−0.016	Fe I?
5303.874	1*n*	5303.845	1.3	−0.029	Fe I?
5181.335	3	5181.330	3.7	−0.005	Fe I?
5173.498	3	5173.487	0.9	−0.011	Fe I
5149.492	1	5149.520	*1.1*	+0.028	Fe I–Fe II
5147.107	2	5147.103	*5.0*	−0.004	Fe I–Fe II
5140.826	3	5140.823	*3.1*	−0.003	Fe I
5097.498	3	5097.492	7.4	−0.006	Fe I
5031.180	1	5031.182	2.2	+0.002	Fe I?
5025.306	3	5025.305	3.6	−0.001	Fe I
5013.914	3	5013.920	4.4	+0.006	Fe I
5007.710	3	5007.734	6.6	+0.024	Fe I?
4992.502	1	4992.480	0.9	−0.022	Fe I?
4980.278	3	4980.296	2.8	+0.018	Fe I
4974.246	0	4974.247	1.6	+0.001	Fe I
4961.040	3	4961.054	3.4	+0.014	Fe I
4944.306	3	4944.287	2.0	−0.019	Fe I
4902.368	1	4902.384	1.7	+0.016	Fe I
4900.816	2	4900.821	2.4	+0.005	Fe I
4861.947	3	4861.952	4.1	+0.005	Fe I
4833.817	1	4833.819	1.7	+0.002	Fe I
4825.357	3	4825.349	6.2	−0.008	Fe I
4821.572	2	4821.601	0.8	+0.029	Fe I
4818.038	1	4818.032	3.9	−0.006	Fe I
4814.365	2	4814.369	2.7	+0.004	Fe I
4805.529	0*n*	4805.55	0.8	+0.02	Fe I?
4768.697	3	4768.700	5.2	+0.003	Fe I
4756.356	1	4756.366	2.3	+0.010	Fe I
4728.160	1	4728.167	3.8	+0.007	Fe I
4716.483	0	4716.508	0.4	+0.025	Fe I?
4712.515	1	4712.497	2.8	−0.018	Fe I
4686.641	0	4686.630	0.3	−0.011	Fe I?
4686.348	0*n*	4686.370	1.1	+0.022	Fe I?
4674.297	2	4674.303	2.8	+0.006	Fe I
4660.920	1*n*	4660.907	4.1	−0.013	Fe I?
4640.958	1	4640.973	3.2	+0.015	Fe I?
4627.532	2	4627.549	2.6	+0.017	Fe I
4605.610	2*n*	4605.594	8.6	−0.016	Fe I
4597.403	2	4597.383	5.0	−0.020	Fe I
4591.502	2*n*	4591.520	7.4	+0.018	Fe I
4590.815	2*n*	4590.793	5.4	−0.022	Fe I
4585.337	1	4585.343	3.0	+0.006	Fe I
4582.297	1*n*	4582.309	3.5	+0.012	Fe I
4581.186	1	4581.196	4.8	+0.010	Fe I
4561.426	2	4561.417	6.1	−0.009	Fe I
4560.892		4560.869	3.1	−0.023	Fe I?
4557.287	2	4557.284	5.7	−0.003	Fe I
4541.319	2	4541.318	3.7	−0.001	Fe I
4533.078	2*n*	4533.046	7.5	−0.032	Fe I?
4517.136	1	4517.154	*6.6*	+0.018	Fe I?
4507.232	1	4507.227	1.4	−0.005	Fe I
4500.652	1*n*	4500.639	*2.9*	−0.013	Fe I
4480.731	1*n*	4480.704	1.0	−0.027	Fe I?
4478.649	1*n*	4478.626	2.7	−0.023	–Fe I
4445.050	0	4445.065	0.4	+0.015	Fe I?
4437.695	1	4437.699	2.7	+0.004	Fe I
4424.608	1	4424.586	5.9	−0.022	–Fe I
4424.061	1	4424.072	5.4	+0.011	Fe I–C II
4420.266	2*n*	4420.287	7.7	+0.021	Fe I
4419.076	2*n*	4419.104	2.7	+0.028	Cr I Fe I
4416.137	0	4416.160	1.5	+0.023	Fe I?
4412.146	1	4412.138	1.8	−0.008	Fe I
4411.914	2*n*	4411.935	12.0	+0.021	|Ti II Fe I?
4378.486	2	4378.512	5.7	+0.026	Fe I
4372.558	1	4372.588	1.4	+0.030	Fe I
4344.928	1	4344.891	8.0	−0.037	–Fe I?
4341.802	0*n*	4341.826	2.8	+0.024	Fe I
4336.122	1	4336.135	0.1	+0.013	Fe I
4335.773	0	4335.783	1.3	+0.010	Fe I?–
4334.928	1*n*	4334.938	3.2	−0.010	Fe I?
4332.432	1	4332.453	1.8	+0.021	Fe I
4331.411	1*n*	4331.442	4.4	+0.031	Fe I —
4288.297	1	4288.268	1.2	−0.029	Fe I?
4276.082	1	4276.103	4.7	+0.021	CH Fe I
4272.528	1	4272.544	10.5	+0.016	Fe I –
4269.730	1*n*	4269.740	17.8	+0.010	Fe I
4269.053	2	4269.034	3.5	−0.019	Fe I
4243.560	2	4243.547	13.7	−0.013	Fe I
4229.406	1	4229.408	7.1	+0.002	Fe I
4223.221	1	4223.236	5.2	+0.015	Fe I
4219.018	1	4219.016	0.4	−0.002	Fe I
4199.557	1*n*	4199.524	0.8	−0.033	Fe I
4197.512	0	4197.508	0.6	−0.004	Fe I
4194.099	1	4194.089	0.5	−0.010	Fe I
4193.600	1	4193.621	3.3	−0.021	Fe I
4192.374	1*n*	4192.400	2.4	+0.026	CN Fe I
4188.729	2*n*	4188.737	28.6	+0.008	Fe I–
4188.300	1*n*	4188.315	3.8	+0.015	Fe I CN?
4185.793	3	4185.779	4.5	−0.014	Fe I
4179.689	1	4179.674	0.6	−0.015	Fe I
4174.208	0*n*	4174.183	0.5	−0.025	Fe I
4160.333	1*n*	4160.368	15.4	+0.035	Fe I –
4158.366	1	4158.376	6.0	+0.010	Fe I
4156.322	1*n*	4156.307	20.4	−0.015	Fe I
4155.914	1	4155.915	8.4	+0.001	Fe I
4148.794	1*n*	4148.783	4.8	−0.011	Fe I Mn I
4139.718	1	4139.732	0.8	+0.014	Fe I
4137.642	1	4137.655	*8.0*	+0.013	Ce II Fe I
4135.039	1*n*	4135.037	9.2	−0.002	Fe I Mn I
4131.146	1*n*	4131.117	11.9	−0.029	Mu I Fe I
4124.490	1	4124.489	7.5	−0.001	Fe I
4124.332	0*n*	4124.358	0.5	+0.026	Fe I?
4110.310	1	4110.299	2.2	−0.011	Ca II–Fe I
4096.695	1	4096.696	9.0	+0.001	Fe I
4095.346	1	4095.356	4.9	+0.010	Fe I
4094.422	1	4094.422	12.0	0.000	Fe I
4081.264	1	4081.262	12.2	−0.002	Fe I
4072.332	0	4072.351	3.7	+0.019	Fe I
4070.010	0	4070.036	2.7	+0.026	Fe I
4069.610	1	4069.610	8.4	0.000	Fe I
4068.898	0	4068.90	0.2	0.00	Fe I
4054.454	1	4054.442	6.9	−0.012	Fe I
4037.136	1*n*	4037.121	10.9	−0.015	Fe I
4036.552	0*n*	4036.567	2.1	+0.015	Fe I
4033.648	1	4033.660	7.7	+0.012	Fe I
4026.770	1	4026.771	2.4	+0.001	Fe I
4022.564	0	4022.536	1.1	−0.028	Fe I?
4022.212	1*n*	4022.226	10.4	+0.014	Fe I
4012.628	1*n*	4012.602	6.2	− 0.026	Ni I?—Fe I
4010.618	1	4010.588	16.7	−0.030	–Fe I
4010.522	1	4010.492	2.7	−0.030	Fe I?
4009.388	1	4009.420	0.9	+0.032	Fe I
4009.240	1*n*	4009.255	1.1	+0.015	Fe I
4003.287	1*n*	4003.275	2.5	−0.012	Mn I–Fe I
4001.212	1*n*	4001.241	1.0	+0.029	Fe I
3996.540	1	3996.546	2.6	+0.006	Fe I
3996.139	1	3996.117	4.0	−0.022	Fe I?
3993.642	1	3993.612	5.0	−0.030	–Fe I
3989.006	1*n*	3988.992	18.8	−0.014	–Fe I
3983.518	1*n*	3983.540	10.3	+0.022	Fe I
3980.008	1	3980.012	6.8	+0.004	Fe I
3970.863	1	3970.843	3.3	−0.020	–Fe I?
3963.438	1	3963.437	12.1	−0.001	Fe I
3962.717	1*n*	3962.722	12.0	+0.005	Fe I
3960.642	1	3960.647	1.4	+0.005	Fe I
3951.638	1*n*	3951.626	7.1	−0.012	Fe I
3943.166	1*n*	3943.182	11.5	+0.016	Fe I
3936.558	1	3936.557	16.2	−0.001	Fe I
3934.356	1*n*	3934.366	35.3	+0.010	Fe I
3932.266	1*n*	3932.254	5.6	−0.012	Fe I
3931.883	0	3931.898	6.1	+0.015	Fe I
3930.876	0*N*	3930.889	10.4	+0.013	Fe I –
3892.302	1	3892.314	10.3	+0.012	Fe I
3818.593	2*n*	3818.620	21.7	+0.027	Fe I
3815.188	1*n*	3815.210	8.9	+0.022	Fe I—CN
*3814.785*	1	3814.784	18.9	−0.001	Fe I
3805.771	3	3805.745	11.3	−0.026	CN—Fe I
3804.501	1	3804.486	7.1	−0.015	CN—Fe I
3801.337	1	3801.371	30.2	+0.034	Fe I —CN
3799.024	0	3799.021	1.1	−0.003	Fe I?
3794.174	0	3794.176	1.6	+0.002	Fe I?
3793.136	1	3793.125	11.3	−0.011	Fe I? CN?
3786.443	3	3786.448	26.6	+0.005	Fe I?
3784.812	1	3784.826	2.3	+0.014	Fe I CN
3784.698	1	3784.675	2.4	−0.023	CN Fe I?
3782.862	3	3782.848	1.2	−0.011	Fe I
3781.300	0	3781.321	1.3	+0.021	Fe I?
3780.966	0	3780.989	2.6	+0.023	Fe I?—CN?
3778.809	1	3778.798	23.9	−0.011	CN Fe I?
3776.838	0	3776.839	0.5	+0.001	Fe I?
3770.548	0	3770.531	3.7	−0.017	Fe I?
3769.310	1*n*	3769.316	2.8	+0.006	Fe I—
3759.597	1*n*	3759.585	13.8	−0.012	Fe I
3711.486	1*n*	3741.479	20.7	−0.007	Fe I
3728.972	2	3728.954	28.1	−0.018	Ni I Fe I
3707.578	1*n*	3707.562	29.2	−0.016	Ti I Fe I
3707.458	2	3707.465	25.4	+0.007	Co I |Fe I
3707.335	1	3707.329	27.6	−0.006	—Fe I
3699.810	1	3699.825	12.0	+0.015	Fe I
3696.548	1*n*	3696.523	17.6	−0.025	Fe I
3695.632	2*n*	3695.652	19.8	+0.020	Fe I
3688.198	1	3688.173	19.0	−0.025	Fe I
3684.552	1	3684.542	14.1	−0.010	Fe I
3680.962	2*n*	3680.944	23.8	−0.018	Fe I
3680.396	2*n*	3680.389	21.2	−0.007	Fe I
3677.503	2	3677.514	26.4	+0.011	Fe I
3672.114	0	3672.124	6.0	+0.010	Fe I
3665.845	1	3665.850	6.3	+0.005	Fe I
3659.214	1	3659.234	3.8	+0.020	Ce II Fe I
3659.094	1*n*	3659.124	0.7	+0.030	Fe I
3651.918	1*n*	3651.921	25.6	+0.003	Fe I—CH
3650.554	2*n*	3650.538	25.5	−0.016	—Fe I
3640.096	1	3640.118	2.3	+0.022	Fe I
3639.964	1*n*	3639.985	2.6	+0.021	Fe I
3639.502	1*n*	3639.525	12.3	+0.023	Fe I —
3639.308	1*n*	3639.332	3.3	+0.024	Fe I
3628.868	1	3628.879	3.4	+0.011	Fe I
3628.620	1	3628.599	12.7	−0.021	—Fe I
3628.414	0	3628.439	2.2	+0.025	Fe I
3625.498	1	3625.501	13.5	+0.003	Fe I
3622.431	3	3622.438	0.8	+0.007	Fe I
3619.942	2*n*	3619.937	13.8	−0.005	Fe I
3618.160	2	3618.187	5.3	+0.027	Fe I
3617.007	2	3617.011	10.6	+0.004	Fe I
3616.857	0	3616.878	0.1	+0.021	Fe I?
3616.722	1	3616.728	1.0	+0.006	Fe I
3615.959	1	3615.962	10.2	+0.003	Fe
3615.814	2	3615.811	2.5	−0.003	F I
3615.518	0	3615.531	0.5	+0.013	Fe I ?
3615.328	0	3615.324	0.8	− 0.004	Fe I
3613.711	1	3613.719	9.7	+0.008	Fe I
*3613.612*	2	3613.605	24.4	−0.007	Fe I
3611.188	1*n*	3611.184	15.0	− 0.004	Fe I
3609.996	1	3609.978	1.2	0.018	Fe I?
3607.780	2*n*	3607.772	1.8	0.008	Fe I
3607.256	1	3607.251	0.8	−0.005	Fe I
3607.102	2*n*	3607.124	1.7	+0.022	Fe I
3606.253	2	3606.251	1.4	−0.002	Fe I
3606.016	0	3606.039	7.5	+0.023	Fe I?
3604.701	2	3604.702	17.3	+0.001	Fe I
3604.090	0*n*	3604.07	0.5	−0.02	Fe I?
3603.956	2	3603.950	12.6	−0.006	Fe I
3603.673	2	3603.691	9.0	+0.018	Fe I
3603.449	1	3603.438	0.9	−0.011	Fe I
3602.898	0	3602.878	0.5	−0.020	Fe I
3601.273	1	3601.284	1.7	+0.011	Fe I
3599.972	2*n*	3599.970	16.2	−0.002	Fe I
3599.842	1	3599.831	1.8	−0.011	Fe I
3596.853	1*n*	3596.859	0.8	+0.006	Fe I
3596.727	0	3596.752	0.4	+0.025	Fe I?
3595.526	1	3595.540	0.3	+0.014	Fe I
3594.312	0	3594.317	2.1	+0.005	Fe I?
3592.354	1	3592.367	1.1	+0.013	Fe I
3589.876	0*n*	3589.882	4.7	+0.006	Fe I?
3584.354	1	3584.383	7.9	+0.029	Fe I
3584.264	1*n*	3584.257	1.5	−0.007	Fe I
3584.110	1	3584.097	10.3	−0.013	Fe I
3583.921	1*n*	3583.911	27.9	−0.010	Fe I
3583.687	2	3583.697	33.2	+0.010	| Fe I —V I
3583.577	1	3583.597	3.9	+0.020	Fe I
3582.970	3*n*	3582.964	5.9	−0.006	Fe I
3582.908	1	3582.877	6.6	−0.031	Fe I?
3582.460	2	3582.437	7.8	−0.023	Fe I
3581.951	1	3581.941	16.4	−0.010	Fe I
3580.402	1	3580.412	12.5	+0.010	Fe I
3579.562	1	3579.562	9.9	0.000	Fe I
3577.490	1*n*	3577.465	13.1	−0.025	Ce II —Fe I
3574.609	2	3574.584	1.5	−0.025	Fe I
3574.256	1	3574.253	12.6	−0.003	Ti I —Fe I
3567.748	1	3567.742	9.5	−0.006	Fe I
3562.269	1*n*	3562.270	10.9	+0.001	Fe I?—Cr I?
3555.736	1	3555.724	3.2	−0.012	Fe I
3539.376	1	3539.371	1.1	−0.005	Fe I
3538.688	1	3538.690	2.3	+0.002	Fe I
3530.976	1	3530.965	7.1	−0.011	Fe I
3528.316	0	3528.324	3.8	+0.008	Fe I?
3525.622	1	3525.618	12.6	−0.004	—Fe I
3519.500	1	3519.505	1.1	+0.005	Fe I
3515.534	1	3515.535	7.7	+0.001	Fe I
3510.682	2	3510.685	15.0	+0.003	Fe I
3506.946	1	3506.938	4.4	−0.008	Fe I
3500.164	2	3500.157	11.1	−0.007	Fe I
3499.271	1	3499.269	6.6	−0.002	Fe I
3498.755	2	3498.749	13.4	−0.006	Fe I
3488.827	2	3488.826	19.5	−0.001	Fe I
3487.138	0	3487.150	3.7	+0.012	Fe I?
3486.142	1	3486.143	10.3	+0.001	Fe I
3483.890	2	3483.884	13.4	−0.006	Fe I
3482.446	1	3482.451	10.6	+0.005	Fe I Fe II
3472.318	0*n*	3472.307	16.4	−0.011	—Fe I
3457.894	1	3457.894	7.5	0.000	Fe I—
3450.743	1	3450.747	2.0	+0.004	Fe I Ti I
3448.606	1*n*	3448.592	6.7	−0.014	Fe I
3444.532	2	3444.518	13.2	−0.014	Fe ]
3435.219	0*n*	3435.246	1.0	+0.027	Fe I?
3430.066	0*n*	3430.083	0.6	+0.017	Fe I?
3429.179	1	3429.148	1.3	−0.031	Fe I?
3425.441	1	3425.446	4.7	+0.005	Fe I Nb II
3423.558	1	3423.534	3.2	−0.024	NH—Fe I?
3422.120	2	3422.127	13.1	+0.007	Fe I
3421.930	1	3421.900	0.6	−0.030	Fe I
3420.250	0*n*	3420.228	2.9	−0.022	Fe I?
3418.905	1	3418.881	16.1	−0.024	Fe I
3416.840	1	3416.869	6.1	40.029	NH Fe I
3414.432	0	3414.403	4.7	−0.029	Fe I?
3408.474	1	3408.505	6.5	+0.031	Fe I
3401.007	1	3400.987	12.2	−0.020	Fe I
3400.662	1	3400.645	10.3	−0.017	Fe I
3398.620	1	3398.612	11.8	−0.008	Fe I| Ti I
3384.946	0*n*	3384.925	3.5	−0.021	Fe I
3384.392	0	3384.425	5.0	+0.033	Fe I?
3381.990	1*n*	3381.993	11.2	+0.003	Fe I
3381.498	1*n*	3381.495	0.9	−0.003	Co I Fe I
3381.132	1	3381.132	16.1	0.000	Fe I —
3380.756	1	3380.752	30.9	−0.004	| Sr II—Fe I
3379.688	1	3379.706	0.9	+0.018	Fe I
3377.971	3	3377.977	23.1	+0.006	Fe I
3377.345	0*n*	3377.361	1.8	+0.016	Fe I —V I
3375.724	1	3375.730	6.8	+0.006	Fe I
3373.300	1	3373.316	17.1	+0.016	Fe I
3371.304	1	3371.295	18.1	−0.009	Fe I
3368.800	1*n*	3368.821	16.5	+0.021	Fe I
3367.660	1	3367.677	17.2	+0.017	NH Fe I
3367.292	1	3367.299	8.9	+0.007	Fe I
3364.402	1	3364.400	10.1	−0.002	Fe I NH
3357.558	1	3357.569	11.6	+0.011	Fe I
3354.512	1	3354.537	7.3	+0.025	Fe I? Ti II?
3340.184	0	3340.178	3.7	−0.006	Fe I
3337.915	1	3337.923	42.3	+0.008	Fe I
3330.206	1	3330.234	13.2	+0.028	Fe I —|NH
3328.589	0	3328.583	2.9	−0.006	Fe I?
3328.470	1*n*	3328.475	12.2	+0.005	Fe I
3324.142	0	3324.150	19.4	+0.008	Fe I?
3316.838	0	3316.851	12.5	+0.013	Fe I —V II
3316.558	1	3316.569	11.4	+0.011	Fe I
3311.200	0*n*	3311.215	15.4	+0.015	Fe I
3310.916	0*n*	3310.918	8.2	+0.002	Fe I
3298.537	1	3298.558	14.8	+0.021	Fe I
3294.621	0*n*	3294.622	11.4	+0.001	Fe I
3282.440	1	3282.447	11.0	+0.007	Fe I
3268.885	1	3268.860	5.2	−0.025	Fe I?
3263.062	1	3263.073	12.9	+0.011	Fe I
3262.878	1	3262.902	21.7	+0.024	Fe I
3261.801	0	3261.817	16.9	+0.016	Fe I
3261.636	0	3261.639	37.2	+0.003	Fe I?—
3260.723	0	3260.692	4.5	−0.031	Fe I?
3260.460	0	3260.472	4.6	+0.012	Fe I?
3259.708	1	3259.713	8.6	+0.005	Fe I
3258.092	1	3258.100	11.4	+0.008	Fe I
3249.844	1	3249.861	13.7	+0.017	Fe I
3249.504	1	3249.535	25.6	+0.031	Fe I—V I
3241.378	1*n*	3241.391	17.9	+0.013	Fe I
3232.656	1	3232.687	16.5	+0.031	Fe I–
3223.480	1*n*	3223.449	17.9	−0.031	Fe I
3216.343	0	3216.359	1.7	+0.016	Fe I?
3205.782	1	3205.783	12.2	+0.001	Fe I
3204.454	0*n*	3204.453	4.7	−0.001	Fe I
3204.306	1	3204.284	17.0	−0.022	— Fe I
3202.958	2	3202.942	4.7	−0.016	Fe I
3198.492	1	3198.487	18.2	−0.005	Fe I
3195.968	1	3195.990	32.2	+0.022	Fe I
3195.235	1*n*	3195.230	26.1	−0.005	Fe I
3192.521	2	3192.534	31.9	+0.013	|| Fe I—CH?
3189.612	0	3189.634	0.5	+0.022	Fe I
3186.276	0	3186.272	9.6	−0.004	Fe I
3184.215	1	3184.210	17.6	−0.005	Fe I
3181.142	0*n*	3181.131	4.1	−0.011	Fe I
3170.978	0	3170.985	14.7	+0.007	Fe I?
3165.280	0	3165.266	21.9	−0.014	Fe I
			Est. Int.		
3159.437	1	3159.436	[−1]	−0.001	Fe I
3158.193	0	3158.191	[−1]	−0.002	Fe I?
3157.293	1	3157.294	[0]	+0.001	Fe I
3146.270	1	3146.301	[0]	+0.031	Fe I
3139.485	1	3139.486	[−1]	+0.001	Fe I
*3135.590*	1*n*	3135.589	[0]	−0.001	Fe I
3134.401	1	3134.396	[1]	−0.005	Fe I?
3132.660	1	3132.635	[1]	−0.025	—Fe I
3123.545	1*n*	3123.561	[1]	+0.016	Fe I
3120.220	2*n*	3120.237	[1]	+0.017	Fe I
3116.502	1*n*	3116.503	[1]	+0.001	Fe I
3115.862	1	3115.883	[0]	+0.021	Fe I
3115.656	2	3115.668	[1]	+0.012	Fe I Cr II
3109.614	1*n*	3109.622	[1]	+0.008	Fe I
3107.322	1*n*	3107.322	[0]	0.000	Fe I
3098.963	1	3098.968	[−1]	+0.005	Fe I
3087.420	1*n*	3087.453	[0]	+0.033	Fe I OH
3081.278	1	3081.247	[2]	−0.031	OH Fe I
3049.564	1*n*	3049.546	[2]	−0.018	Fe I
3049.356	1*n*	3049.349	[3]	−0.007	Fe I
3038.334	0*n*	3038.312	[3]	−0.022	Fe I
3012.942	1*n*	3012.937	[3]	−0.005	Fe I
3011.883	2	3011.88	[0N]	0.00	Fe I
2995.256	0	2995.260	[3]	+0.004	Fe I
2985.750	0	2985.73	[1]	−0.02	Fe I
2979.867	0	2979.88	[1]	+0.01	Fe I
2978.060	1	2978.055	[3]	−0.005	Fe I
2975.298	0	2975.278	[3]	−0.020	–Fe I
2971.776	0	2971.77	[1]	−0.01	Fe I
2963.518	0*n*	2963.52	[3N]	0.00	Fe I? |Cr II
2962.585	1*n*	2962.59	[−1]	0.00	Fe I
2958.462	1	2958.45	[0N]	−0.01	Fe I
2947.116	0	2947.04	[−1]	−0.08	W I—Fe I?
2946.095	1	2946.08	[−1]	+0.02	Fe I
2945.702	1	2945.65	[−1]	−0.05	Fe I

## Footnotes

1 Figures in brackets indicate the literature references on page 3.
